# Efficacy and Safety of the Chinese Patent Medicine Yuquan Pill on Type 2 Diabetes Mellitus Patients: A Systematic Review and Meta-Analysis

**DOI:** 10.1155/2021/2562590

**Published:** 2021-12-02

**Authors:** Sihan Peng, Ziyan Xie, Xiyu Zhang, Chunguang Xie, Jian Kang, Haipo Yuan, Gang Xu, Xiangeng Zhang, Ya Liu

**Affiliations:** ^1^Hospital of Chengdu University of Traditional Chinese Medicine, Chengdu, Sichuan, China; ^2^Chengdu University of Traditional Chinese Medicine, Chengdu, Sichuan, China; ^3^Sichuan Nursing Vocational College, Chengdu, Sichuan, China

## Abstract

**Background:**

Yuquan Pill (YQP), a Chinese patent medicine for the treatment of diabetes, is widely used in the treatment of diabetes and its complications in China. However, the efficacy of YQP on type 2 diabetes mellitus (T2DM) has not been completely assessed. The aim of this study is to evaluate the efficacy and safety of YQP in the treatment of T2DM.

**Materials and Methods:**

We systematically searched 9 databases for specific keywords from inception to Oct 2021. We included randomized controlled trials (RCTs) involving YQP in the treatment of T2DM without language limitation. The study conformed to the Cochrane Handbook and Review Manager software was used for data analysis. The weighted mean differences (WMDs) and 95% confidence intervals (CIs) were used to measure treatment effects.

**Results:**

The final analysis included 10 publications. Analysis showed that the combination of YQP and conventional treatment was more effective than conventional treatment alone with regard to the levels of fasting blood glucose (WMD = −0.83; 95% CI [−1.01,−0.66]; *p* < 0.00001), two-hour postprandial glucose (WMD = −1.40; 95% CI [−1.49,−1.31]; *p* < 0.00001), glycosylated hemoglobin (WMD = −0.87; 95% CI [−1.26, −0.49]; *p* < 0.00001), total cholesterol (WMD = −0.50; 95% CI [−0.61, −0.39]; *p* < 0.00001), c-reactive protein (WMD = −0.58; 95%CI [−0.88, −0.28]; *p*=0.0002), and overall effective rate (RR = 1.21; 95% CI [1.12, 1.31]; *p* < 0.00001).

**Conclusion:**

Evidence suggested that YQP might improve glucose and lipid metabolism and inflammation in patients with T2DM. Serious adverse events were not reported. The quality of the evidence analyzed was low and therefore our results should be interpreted with caution. More high-quality RCTs are now needed to verify these findings.

## 1. Introduction

Type 2 diabetes mellitus (T2DM) is a chronic metabolic disease that is characterized by elevated levels of blood glucose. The pathophysiology of T2DM is characterized by insulin resistance accompanied by reduced insulin secretion due to the dysfunction of pancreatic *β* cells [1]. Over the past three decades, the number of people living with diabetes has increased more than double globally. Diabetes has now become a major public health issue across the entire world [2–4]. Moreover, diabetes and its complications have a significant economic impact on individuals, families, and countries [5]. The global prevalence of diabetes is estimated to be 9.3% (approximately 463 million people), with T2DM accounting for more than 90% of all diabetics [6, 7]. Insulin resistance and long-term hyperglycemia will inevitably lead to changes in various metabolic and cellular functions, such as dyslipidemia, increased blood pressure, endothelial dysfunction, and increased platelet reactivity and oxidative stress. These factors can cause chronic inflammation, thereby damaging blood vessels and accelerating the occurrence and development of diabetic microvascular and macrovascular complications [8–12]. Although routine hypoglycemic drugs are widely used, these still fail to effectively prevent the progression of diabetes and may cause side effects such as gastrointestinal reactions (nausea, vomiting, and diarrhea), dizziness [13], and hypoglycemia [14]. Thus, the treatment of T2DM has become a significant hotspot for medical research, particularly with regard to developing new treatments for T2DM.

**Figure 1:**
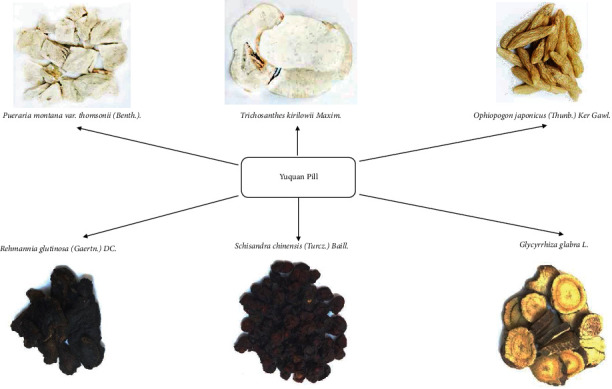
The composition of Yuquan Pill.

Traditional Chinese medicine (TCM) has a long history in the treatment of diabetes and has been associated with remarkable curative effects. TCM plays an important role in the management of diabetes in China [15] and is believed to relieve diabetes by comprehensively regulating the qi and blood balance in human body. As a representative Chinese patent medicine, YQP has been approved by the China Food and Drug Administration (CFDA) (approval number: Z51021085) for the treatment of diabetes and its complications. YQP comprises the following herbs: *Pueraria montana* var. Thomsonii (Benth.) (*gě gēn*), *Trichosanthes kirilowii* Maxim. (*tiān huā fěn*), *Ophiopogon japonicus* (Thunb.) Ker Gawl. (*mài dōng*), *Rehmannia glutinosa* (Gaertn.) DC. (*dì huáng*), *Schisandra chinensis* (Turcz.) Baill. (*wǔ wèi zǐ*), and *Glycyrrhiza glabra L*. (*gān cǎo*). According to the basic theory of TCM, T2DM is triggered by yin deficiency. YQP has the efficacy of nourishing yin, moistening dryness, and promoting fluid, which can treat T2DM in essence. The composition and details of YQP are generalized in Figure 1 and Table 1.

Since its approval, YQP has become one of the most commonly used Chinese patent medicines to treat diabetes and its complications. The Guidelines for the *Prevention and Treatment of Diabetes by Traditional Chinese Medicine (2011 Edition)* issued by the Chinese Medical Association recommends YQP as the prescription for the treatment of diabetes [16]. Studies have shown that YQP can improve insulin sensitivity, regulate glucose and lipid metabolism, and improve macrovascular disease and microvascular disease in diabetic patients [17–19]. YQP has also been shown to protect endothelial cells, alleviate inflammatory responses, and reduce the serum levels of vascular endothelial growth factor (VEGF) [20–22]. An increasing number of clinical studies have reported that YQP may effectively improve the clinical symptoms and indicators of T2DM [23–26]. However, to the best of our knowledge, there has been no evaluation that has systematically analyzed the efficacy and safety of YQP and whether this drug represents an ideal form of alternative therapy. In the present study, we used evidence-based methods to evaluate the efficacy and safety of YQP for the treatment of T2DM. Our aim was to provide more robust scientific evidence for clinicians, researchers, and policy makers.

## 2. Methods

### 2.1. Database and Search Strategies

The protocol for this systematic review was registered in the International Prospective Register of Systematic Reviews (PROSPERO) with a registration number CRD42021261805.

This review was designed and performed in accordance with the guidelines of Preferred Reporting Items for Systematic Review and Meta-Analysis Protocols (PRISMA-P) 2015 [27]. All reviewers received relevant training to grasp the background, purpose, and process of the review.

We systematically searched 9 databases for specific keywords from inception to Oct 2021, including PubMed, Web of Science, the Cochrane Library, the China National Knowledge Infrastructure (CNKI), China Biology Medicine Disc (Sino Med), the Wan Fang Database, and the VIP information resource integration service platform (CQVIP). YQP (approval number: Z51021085) is a commonly used Chinese patent medicine for the treatment of diabetes in China. In order to avoid missing unpublished clinical trials for patent reasons, we additionally searched the patent database. A detailed retrieval of domestic and foreign patent information was carried out in Patent Search and Analysis of National Intellectual Property Administration (http://pss-system.cnipa.gov.cn/sipopublicsearch/portal/uiIndex.shtml). Google scholar was also carefully searched to find missing publications. We included all RCTs that were associated with YQP for T2DM without language limitation. The search terms were as follows: “Yuquan Pill,” “Yuquan wan,” “Yuquan,” “Yu-Quan Pill,” “yuquan pill,” “Diabetes Mellitus,” “Diabetes Insipidus,” “Diet, Diabetic,” “Prediabetic State,” “Glycation End Products, Advanced,” “Gastroparesis,” and “Glucose Intolerance.” Meanwhile, we also carefully studied the references of the relevant literature for more available studies.

### 2.2. Inclusion and Exclusion Criteria

Only randomized controlled trials (RCTs) that assessed the effects of YQP for T2DM were selected. Reviews, retrospective study, and animal experiments were excluded. For participants, we only included adult patients with a definite diagnosis of T2DM, without limitations relating to gender, regions, and ethnicity. According to the American Diabetes Association's diabetes guidelines [28], the diagnostic criteria for T2DM are as follows: fasting blood glucose (FBG) ≥126 mg/dL (7.0 mmol/L) or 2-hour postprandial blood glucose (2hPG) ≥200 mg/dL (11.1 mmol/L) during Oral glucose tolerance test (OGTT) or glycosylated hemoglobin (HbA1c) ≥6.5% (48 mmol/mol) or in a patient with classic symptoms of hyperglycemia or hyperglycemic crisis, a random plasma glucose ≥200 mg/dL (11.1 mmol/L). In terms of types of interventions, the treatment groups were treated with YQP, including pills, decoction, and granules, regardless of frequency and duration. The control group received conventional diabetes medicine or placebo treatment. It was worth mentioning that the studies that adopted multiple interventions or that YQP was not the primary intervention had been excluded. As this research aims to systematically evaluate the effects of YQP in the treatment of T2DM, the primary outcomes of the present study were FBG, 2hPG, and HbA1c. The secondary outcomes were total cholesterol (TC), c-reactive protein (CRP), and overall effective rate, while the safety was measured by adverse effects.

### 2.3. Data Collection and Analysis

#### 2.3.1. Study Selection

Study selection was independently done by two investigators (JK and HY), based on the titles and abstracts to select for appropriate publications. EndNote V.X9 software was used for literature management. Any disagreements were submitted to a senior investigator (GX).

#### 2.3.2. Data Extraction and Management

The following information was independently extracted by two reviewers (JK and GX): general information, including the first author, journal, publication time, and country; participant characteristics, such as sample size, gender, mean age, and duration of disease; interventions of treatment and control groups; outcomes including FBG, 2hPG, HbA1c, TC, CRP, and overall effective rate. The information was cross-checked by two investigators, and any disagreements were resolved by a third reviewer (SP).

#### 2.3.3. Assessment of Risk of Bias

Two independent investigators (JK and GX) assessed the risk of bias in the light of the Cochrane Collaboration's Risk of Bias tool [29]. The grades were rated as “low,” “high,” or “unclear” risk of bias, based on the following items: random sequence generation, allocation concealment, incomplete data, blinding, selective reporting, and other sources of bias. Any differences were discussed and resolved with a third researcher (HY).

#### 2.3.4. Data Analysis

We used Review Manager (version 5.3) software to analyze all data to evaluate the effect of YQP on T2DM patients from the aspects of blood glucose, blood lipid, and so on. Continuous variables, including FBG, 2hPG, HbA1c, TC, and CRP, were evaluated by weighted mean differences (WMDs) and 95% confidence intervals (CIs). Dichotomous data, such as overall effective rate and risk ratio (RR) with 95% CI, was used to measure the treatment effect. When important data was incomplete in the reported literature, we contacted the authors by various means to get more relevant information.

#### 2.3.5. Subgroup Analysis

The heterogeneity was assessed by the *I*^2^ value. If the *I*^2^ value exceeds 50%, it means that there is significant statistical heterogeneity [30]. Subgroup analysis was constructed to explore the potential causes of heterogeneity. The subgroup analysis focused on the following factors: different ages (≥50 y or < 50 y), different control groups (metformin or other treatments), durations of T2DM (≥10 y or < 10 y) or different regions, and so on.

#### 2.3.6. Sensitivity Analysis

When the outcomes were unstable, sensitivity analysis was carried out by removing studies with high risk of bias and recalculating the pooled data to evaluate the robustness of merged results.

#### 2.3.7. Assessment of Publication Bias

Due to insufficient included studies, there was no evaluation of publication bias conducted in this study.

## 3. Results

### 3.1. Literature Search

**Figure 2:**
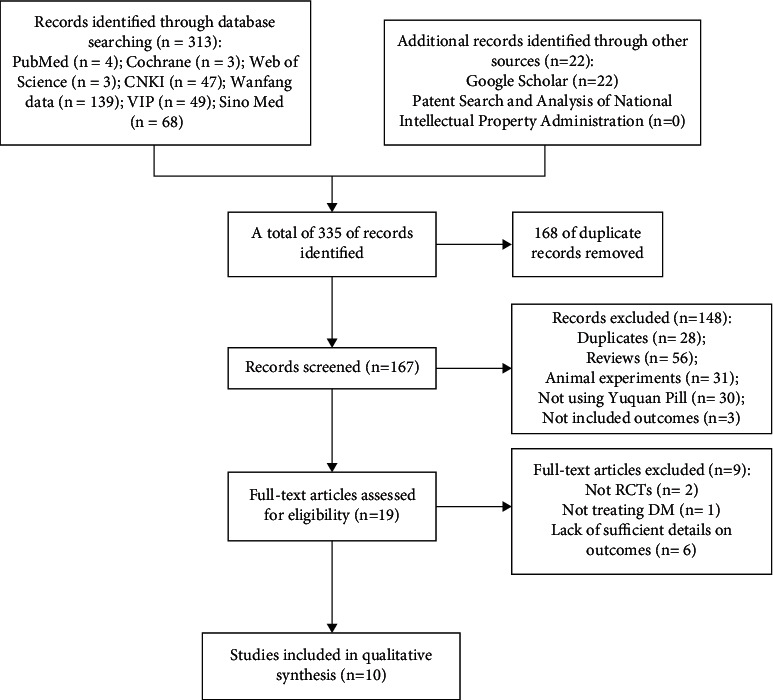
Flow diagram of studies selection process.

Figure 2 shows the process used to select studies for analysis. A total of 335 relevant literature articles were initially identified by our database searches; 168 publications were excluded due to duplicate findings. Of the remaining 167 articles, some were excluded because they failed to meet our specific inclusion criteria. For example, they were reviews or involved animal experiments, and finally there were 19 literatures left. Then, after carefully reading the full texts of these 19 articles, we removed non-RCTs [31, 32], RCTs without treating DM [33], and the articles lacking sufficient details on outcomes [34–39]. At last, 10 clinical trials [40–49] were included in the meta-analysis.

### 3.2. Characteristics of the Included Studies

A total of 871 patients were included in the 10 studies, 455 in treatment groups and 416 in control groups. The mean age of the patients described in these 10 articles ranged from 41.70 ± 11.05 years [43] to 69.30 ± 5.35 years [42]. The intervention measure used for all treatment groups was the administration of YQP in combination with conventional therapy. Of the 10 articles, 9 articles referred directly to T2DM [41–49], while one article referred specifically to diabetic nephropathy [40]; we included this latter article because diabetic nephropathy was a common complication of diabetes. All 10 studies were carried out in China and were published between 2005 and 2021. The shortest and longest treatment duration were 1 month [42, 49] and 3 months [41, 43, 45, 48], respectively.  Table 2 shows the detailed characteristics of the 10 studies that underwent final analysis herein.

### 3.3. The Risk of Bias in the Analyzed Studies

**Figure 3:**
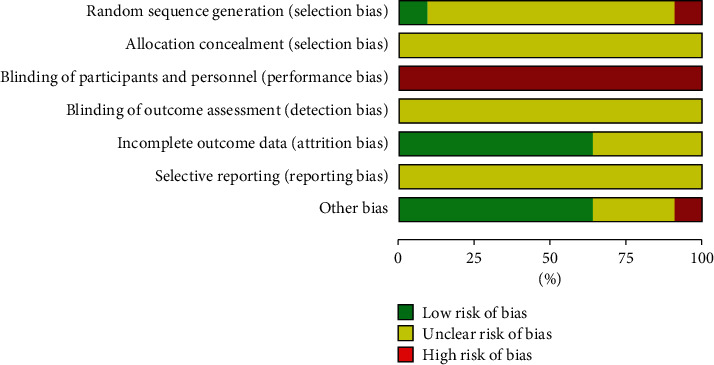
Risk of bias graph.

**Figure 4:**
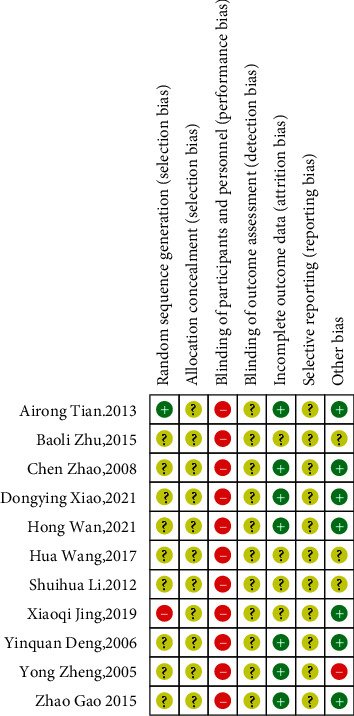
Risk of bias summary.

We investigated the risk of bias for all the articles included in our analysis. All the study items were randomly divided into a YQP group and a control group. The randomization of treatments was not consistent when compared across the 10 articles; only one study [42] was classified as low risk of bias because it was randomized using a random number table; another study [48] did not include randomization and was therefore associated with a high risk of bias; the other 8 studies did not mention the specific randomization method used; the risk of bias for these papers was therefore determined as being ‘unclear.' None of the RCTs reported allocation concealment. In terms of performance bias, none of the 10 articles included in this analysis reported the blinding of the participants or researchers. Therefore, we classified all 10 studies as having a high risk of bias in this domain. With regard to other biases, one study [40] did not report the treatment duration; therefore the risk was classified as being high. The complete and detailed analysis for the risk of bias is presented in Figures 3 and 4.

### 3.4. Outcomes

#### 3.4.1. Fasting Blood Glucose

**Figure 5:**
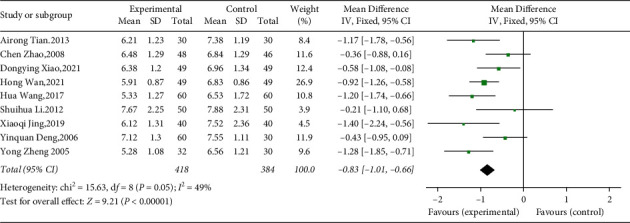
Forest plot for FBG.

Nine studies involving 802 diabetic patients provided data on FBG before and after intervention between treatment and control groups [40–48]. Using fixed effects model, the results suggested that YQP plus conventional treatment might significantly reduce FBG in patients with T2DM (WMD = −0.83; 95% CI [−1.01,−0.66], *p* < 0.00001; see Figure 5). And the heterogeneity was low (*χ*^2^ = 15.63; *p*=0.05,I^2^ = 49%; see Figure 5). In subgroup analysis, there was no significant difference between subgroups of different ages (*p*=0.14)and different control treatments (*p*=0.31) (Table 3, Supplementary Materials 1 and 2).

#### 3.4.2. Two-Hour Postprandial Glucose

**Figure 6:**
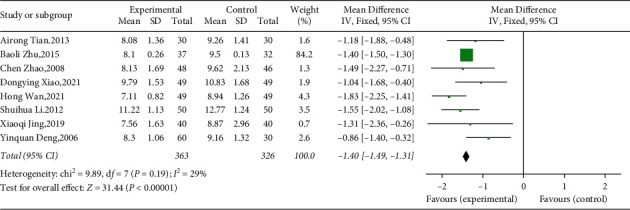
Forest plot for 2hPG.

Eight studies (including 689 patients) evaluated the effect of YQP on 2hPG [42–49]. Results from pooled data analysis implied a significant decrease in 2 hPG with low heterogeneity (WMD = −1.40; 95% CI [−1.49, −1.31]; *p* < 0.00001; heterogeneity: *p*=0.19, I^2^ = 29%, fixed effects model; see Figure 6). In subgroup analysis, there was no significant difference between subgroups of different ages (*p*=0.19), different control treatments (*p*=0.64), and different courses of treatment (*p*=0.95) (Table 3, Supplementary Materials 3–5).

#### 3.4.3. Glycosylated Hemoglobin

**Figure 7:**
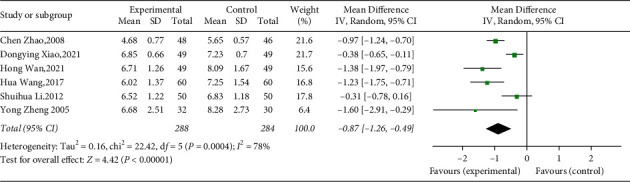
Forest plot for HbA1c.

A total of 6 studies including 572 participants reported HbA1c levels [40, 41, 44–47]. According to the random effects model, the results showed that YQP combined with conventional treatment could reduce the HbA1c levels of T2DM (WMD = −0.87; 95% CI [−1.26, −0.49]; *p* < 0.00001; see Figure 7). We found a significant heterogeneity among these researches (*χ*^2^ = 22.42, *p*=0.0004, and *I*^2^ = 78%; see Figure 7); therefore, subgroup analysis was performed. Subgroup analyses according to different ages, control treatments, and durations of disease showed no significant difference in intervention effect between groups (*p* for interaction = 0.11, 0.74, and 0.26, resp.), and the significant heterogeneity was still observed (Table 3, Supplementary Materials 6–8).

#### 3.4.4. Total Cholesterol

**Figure 8:**
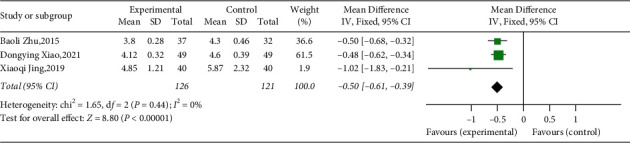
Forest plot for TC.

Three studies involving 247 T2DM evaluated the effect of adding YQP to conventional treatment on TC levels [45, 48, 49]. The combination therapy might significantly reduce the TC levels in T2DM patients compared with control group (WMD = −0.50; 95% CI [−0.61, −0.39]; *p* < 0.00001, fixed effects model; see Figure 8) and with low heterogeneity (*χ*^2^ = 1.65, *p*=0.44, and I^2^ = 0%; see Figure 8). In subgroup analysis, there was no significant difference between subgroups of different ages (*p*=0.20) and different courses of treatment (*p*=0.97) (Table 3, Supplementary Materials 9–10).

#### 3.4.5. C-Reactive Protein

**Figure 9:**
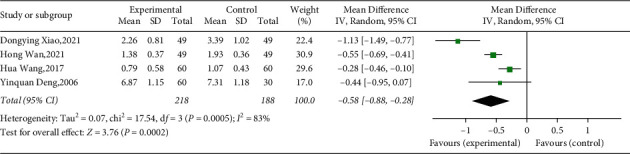
Forest plot for CRP.

Four studies explored CRP between treatment group and control group [41, 43–45]. All the studies involving 406 T2DM patients showed that YQP plus conventional treatment might significantly reduce CRP (WMD = −0.58; 95% CI [−0.88,−0.28]; *p*=0.0002, random effects model; see Figure 9). Significant heterogeneity was observed (*χ*^2^ = 17.54, *p*=0.0005, and I^2^ = 83%; see Figure 9). Subgroup analyses according to different ages, safety, and regions showed no significant difference between groups (*p* for interaction = 0.84, 0.05, and 0.81, resp.) (Table 3, Supplementary Materials 11–13).

#### 3.4.6. Overall Effective Rate

**Figure 10:**
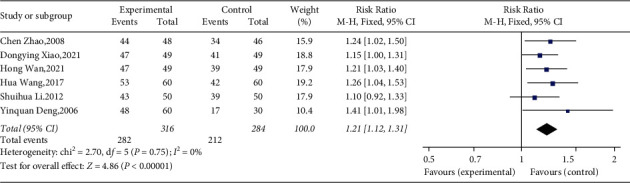
Forest plot for overall effective rate.

The overall effective rate was divided into significant effective, effective, and ineffective according to the degree of improvement of clinical symptoms and related indicators (mainly refers to blood glucose level, including FBG, 2hPG, and HbA1c). Six researches that involved 600 patients mentioned overall effective rate as outcome [41, 43–47]. Since the absence of substantial heterogeneity (*χ*^2^ = 2.70, *p*=0.75, and *I*^2^ = 0%; see Figure 10), we used the fixed effects model for statistical analysis. The results of the meta-analysis showed that YQP might result in a significant increase in overall effective rate compared with conventional treatment (RR = 1.21; 95% CI [1.12, 1.31]; *p* < 0.00001; see Figure 10). Subgroup analyses by different ages, control treatments, courses of treatment, and regions showed no significant difference in effect size (*p* for interaction = 0.90, 0.79, 0.27, and 0.50, resp.) (Table 3, Supplementary Materials 14–17).

#### 3.4.7. Adverse Effects

Adverse effects were reported in 7 of 10 studies. Three of them reported that no adverse effect was found [42, 43, 48]. Four studies [41, 44, 45, 49] reported the occurrence of general adverse effects. The most common adverse effect among these studies was abdominal discomfort [41, 44, 45, 49]. Headache, dizziness, nausea, and hypoglycemia were also reported to occur in the researches. No adverse effects were reported in the remaining studies.

## 4. Discussion

### 4.1. Main Results

With the continuous development and wide application of evidence-based medicine, systematic reviews and meta-analyses have become a recognized and widely accepted research method. Such studies can facilitate the evaluation of clinical evidence and serve as a strong foundation for evidence-based decision making [50]. However, until now, there has been no specific evaluation of the clinical evidence related to the use of YQP for the treatment of T2DM.

A total of 335 relevant articles were retrieved in our initial database searches. Ten of these articles were included in our final meta-analysis after eliminating duplicate articles and applying our specific exclusion criteria. Data analysis demonstrated that, compared to conventional treatment alone, the coadministration of YQP and conventional medicine may be more effective with regard to exerting effects on FBG, 2hPG, HbA1c, TC, and CRP; there was also evidence for an improvement in the overall effective rate. Our analyses also showed that there was evidence for improvements in glucose and lipid metabolism and the inflammation associated with T2DM, at least to some extent. Next, we divided the participants into subgroups according to their specific characteristics (such as age and region) and differences with regard to control treatment and the duration of treatment. Comprehensive subgroup analysis was then performed on the basis of these variables to explain or reduce the observed levels of heterogeneity. Although a series of subgroup analyses were conducted in the present study, we identified high levels of heterogeneity with regard to HbA1c and CRP data. The combination of effect sizes using a random effects model did not reduce the observed heterogeneity or identify the source of such heterogeneity. It was considered that it might be related to the difference in origin, quality, and water content of the Chinese herbs [51, 52] and further analysis could not be conducted due to no specific details in the article.

When considering the statistical heterogeneity of meta-analysis, it is important to consider two different components: clinical heterogeneity and methodological heterogeneity. In our meta-analysis, it is possible that different T2DM participants and different intervention measures may lead to a certain extent of clinical heterogeneity. Methodological heterogeneity may be caused by differences in trial design and poor methodological quality, such as the inappropriate use of blinding methods and hidden differences in allocation. In the present study, our subgroup analysis was based on a range of factors, including age, course of disease, intervention measures, and the treatment duration; these analyses involved post hoc analysis, thus reducing the reliability of our subgroup analysis.

### 4.2. Certainty of the Evidence

**Figure 11:**
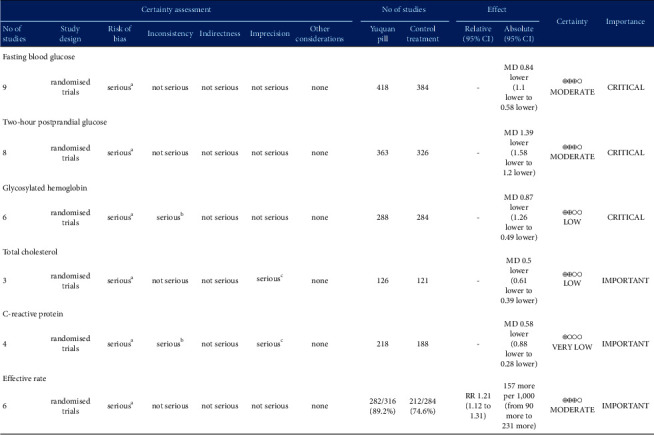
Certainty of evidence: Yuquan Pill compared to control treatment for T2DM. CI, confidence interval; MD, mean difference; RR, risk ratio. ^a^ Randomization allocation and the blinding are unclear in articles. ^b^ There is serious heterogeneity among the studies included in the analysis of this outcome. ^c^ Results are imprecise since the study included relatively few studies and few patients.

We used GRADEpro software to assess the certainty of the evidence that was analyzed in this study.  Figure 11 showed that the FBG, 2hPG, and the effective rate had a moderate quality of evidence, while HbA1c and TC in RCTs had a low certainty of evidence, and CRP had a very low certainty of evidence. The observed reduction in the certainty of evidence was mainly attributed to the high risk of bias in the studies analyzed, inconsistency between the included studies, and the imprecision of the findings. Specifically, this is due to the low methodological quality of the included studies, the high heterogeneity of some studies, and the small sample size of some studies. The poor methodological quality of some of the included studies was indicated by unclear randomization allocation and blinding strategies. As a direct result of these factors, our current findings should be considered cautiously for clinical practice. Additional and more standardized RCTs are now needed to fully validate the effects of YQP on T2DM.

### 4.3. Strengths and Limitations

Our study has several advantages. For example, this is the first systematic review to investigate the efficacy and safety of YQP for the treatment of T2DM. Furthermore, we used a wide range of search terms to conduct a comprehensive and systematic search of established national and international databases. When missing or unclear data were evident, we actively contacted the authors to obtain relevant information. Secondly, in order to provide a comprehensive description of the use of YQP in the treatment of T2DM, this study was conducted in strict accordance with established methodology for systematic reviews. The results were carefully interpreted to ensure accuracy and avoid misleading conclusions. Thirdly, this systematic review and meta-analysis described and evaluated the present clinical trials on YQP for T2DM, filling the blank in existing knowledge. We found that, compared with conventional treatment, the combined use of YQP and conventional medicine could improve glucose and lipid metabolism and inflammation, associated with T2DM. The results of this study provide new treatment options for T2DM. YQP may improve the inflammatory condition of diabetes, which is the pathological basis and key factor of multiple complications of diabetes. It has far-reaching influence, extensive research prospects, and important research significance, and it is worth further investigation. Although our current research is limited, we believe that, with the continuous development and promotion of traditional Chinese medicine, the importance of YQP for T2DM will become increasingly prominent. We hope that our study may provide new directions, ideas, and methods for the study of T2DM.

Although we made every effort to be rigorous and accurate in our research process, some limitations should be taken into account. First, the number of studies included in this meta-analysis was small and of low quality; these factors led to a low degree of certainty in terms of evidence. Consequently, our results should be interpreted with caution. Secondly, there were some defects in the included articles with regard to RCT design. Certain methodologies related to random sequence generation, the allocation concealment, and blinding were generally poor. Only one study [42] clearly reported the generation of random sequences. Furthermore, none of the RCTs involved allocation concealment or the blinding of participants or researchers. This may have led to the overestimation of efficacy. Thirdly, since patients were only recruited from Chinese hospitals, our study may not be globally representative and lacks clinical applicability. Whether YQP can be applied to other ethnic groups remains to be elucidated. In addition, the safety of YQP remains unclear because some studies did not report adverse events; thus, adverse events could not be systematically collated and analyzed. In subgroup analyses, the cutoff points for age and duration of disease are mainly based on related studies, and more biological basis is needed. Although we performed subgroup analyses for each outcome, the source of heterogeneity was not fully identified. Due to the limited number of studies included in this meta-analysis, we were unable to conduct meta-regression analysis to further explore the source of heterogeneity. Furthermore, we were unable to apply funnel plots to evaluate publication bias.

## 5. Conclusion

In conclusion, this systematic review and meta-analysis evaluates the efficacy and safety of YQP on T2DM for the first time. We showed that, compared with conventional treatment, the combined administration of YQP and conventional medicine may improve glucose and lipid metabolism and the inflammatory conditions in patients with T2DM. However, due to the poor quality of these studies, the evidence remains very uncertain. Therefore, our results should be interpreted with caution and applied cautiously in clinical practice. In the future, more multicenter, large-scale, and high-quality RCTs will be needed to fully determine the clinical efficacy and safety of YQP for the treatment of T2DM.

## Figures and Tables

**Table 1:** Details of Yuquan Pill.

Chinese herbs	Latin name	Family	Part of herbs	Function in Chinese medicine
Ge Gen (gě gēn)	*Pueraria montana* var. *Thomsonii* (Benth.).	Fabaceae	Root	To promote fluid, relieve thirst, and clear heat
Tian Hua Fen (tiān huā fěn)	*Trichosanthes kirilowii* Maxim.	Cucurbitaceae	Root	To clear heat and promote fluid
Mai Dong (mài dōng)	*Ophiopogon japonicus* (Thunb.) Ker Gawl.	Asparagaceae	Root	To nourish yin, moisten lung, and promote fluid
Di Huang (dì huáng)	*Rehmannia glutinosa* (Gaertn.) DC.	Plantaginaceae	Root	To nourish yin, promote fluid, and clear heat
Wu Wei Zi (wǔ wèi zǐ)	*Schisandra chinensis* (Turcz.) Baill.	Schisandraceae	Fructus	To promote fluid and nourish lung and kidney
Gan Cao (gān cǎo)	*Glycyrrhiza glabra* L.	Fabaceae	Root and rhizome	To replenish qi, tonify spleen, moisten lung, and promote fluid

**Table 2:** Characteristics of the studies included in this meta-analysis.

Author, year	Age (T/C)	Number (T/C)	Duration of disease (T/C)	Gender (F/M)	Intervention (T)	Intervention (C)	Course	Outcomes	Region
Zheng and Huang, 2005	46.84 ± 12.52/47.59 ± 11.98	32/30	11.25 ± 9.72 y/11.82 ± 8.13 y	32/30	YQP (8 pills, tid)+metformin/gliclazide	Metformin/gliclazide	NR	①③	Hefei, Anhui
Deng et al., 2006	50 ± 11.6/49.8 ± 12.1	60/30	3.68 ± 2.56 y/3.43 ± 2.12 y	51/39	YQP (6 g, qid)+acarbose (50 mg, tid)	Acarbose (50 mg, tid)	12 w	①②⑤⑥⑦	Hangzhou, Zhejiang
Zhao et al., 2008	41.1 ± 10.4/42.3 ± 11.7	48/46	5.2 ± 3.3 y/4.9 ± 3.2 y	53/41	YQP (8 pills, tid)+metformin (0.25 g, tid)	Metformin (0.25 g, tid)	8 w	①②③⑥	Changsha, Hunan
Li and Wu, 2012	51.5 ± 4/53.5 ± 5.5	50/50	4.8 ± 0.9 y/5.1 ± 1.2 y	53/47	YQP (6 g, qid)+metformin (0.5 g, bid)	Metformin (0.5 g, bid)	6 w	①②③⑥	Weinan, Shanxi
Tian, 2013	49.1 ± 4.7/48.3 ± 5.4	30/30	7.1 ± 0.6 y/6.6 ± 1 y	28/32	YQP decoction + hypoglycemic drugs	Hypoglycemic drugs	4 w	①②⑦	Zhengzhou, Henan
Zhu, 2015	52.3 ± 5.1/53.1 ± 3.4	37/32	9.5 ± 2.1 y/10.5 ± 1.5 y	41/28	YQP (6 g, qid)+acarbose (50 mg, tid)	Acarbose (50 mg, tid)	4 w	②④⑦	Wulanchabu, Neimenggu
Wang, 2017	56.27 ± 6.6/57.31 ± 4.2	60/60	5.56 ± 2.35 y/5.45 ± 2.56 y	66/54	YQP (6 g, qid)+metformin enteric capsules (0.5 g, tid)+ insulin	Metformin enteric capsules (0.5 g, tid)+ insulin	12 w	①③⑤⑥⑦	Tianjin
Jing et al., 2019	46.87 ± 7.14/46.95 ± 7.54	40/40	NR	55/25	YQP (6 g, qid)+metformin/gliclazide/insulin	Metformin/gliclazide/insulin	12 w	①②④⑦	Taiyuan, Shanxi
Xiao et al., 2021	68.9 ± 5.2/69.7 ± 5.5	49/49	5.9 ± 1.8 y/5.6 ± 1.6 y	57/41	YQP (6 g, qid)+saxagliptin (5 mg, qd)	Saxagliptin (5 mg, qd)	12 w	①②③④⑤⑥⑦	Nanyang, Henan
Wan et al., 2021	49.59 ± 5.37/49.65 ± 6.29	49/49	5.14 ± 0.92 y/5.24 ± 0.84 y	58/40	YQP (6 g, qid)+dapagliflozin (10 mg, qd)	Dapagliflozin (10 mg, qd)	12 w	①②③⑤⑥⑦	Zhengzhou, Henan

T: treatment group; C: control group; YQP : Yuquan Pill; F: female; M: male; NR: not reported; Y: year; W: week; ①: fasting blood glucose; ②: two-hour postprandial glucose; ③: glycosylated hemoglobin; ④: total cholesterol; ⑤: c-reactive protein; ⑥: overall effective rate; ⑦: adverse effects.

**Table 3:** Subgroup analysis for outcomes.

	Number of comparisons	Results	*p* value for overall effect	*I* ^2^	*p* value for subgroup difference
**FBG**		WMD (95%CI)			
All comparisons	9	−0.84 [−1.10, −0.58]	<0.00001	49%	
Age					0.14
<50 y	5	−0.94 [−1.17, −0.71]	<0.00001	49%	
≥50 y	4	−0.67 [−0.95, −0.38]	<0.00001	47%	
Different control treatment					0.31
Metformin	3	−0.68 [−1.03, −0.34]	0.0001	67%	
Other treatments	6	−0.89 [−1.10, −0.68]	<0.00001	42%	
**2hPG**					
All comparisons	8	−1.39 [−1.58, −1.20]	<0.00001	29%	
Age					0.19
<50 y	4	−1.60 [−1.91, −1.29]	<0.00001	0%	
≥50 y	4	−1.38 [−1.47, −1.29]	<0.00001	43%	
Different control treatment					0.64
Metformin	2	−1.53 [−1.93, −1.13]	<0.00001	0%	
Acarbose	2	−1.38 [−1.48, −1.29]	<0.00001	73%	
Other treatments	4	−1.49 [−1.79, −1.19]	<0.00001	43%	
Course of treatment					0.95
<8 w	3	−1.39 [−1.49, −1.30]	<0.00001	0%	
≥8 w	5	−1.38 [−1.65, −1.12]	<0.00001	56%	
**HbA1c**					
All comparisons	6	−0.87 [−1.26, −0.49]	<0.00001	78%	
Age					0.11
<50 y	3	−1.09 [−1.38, −0.80]	<0.00001	10%	
≥50 y	3	−0.61 [−1.12, −0.11]	0.02	78%	
Different control treatment					0.74
Metformin	3	−0.84 [−1.31, −0.37]	0.0005	74%	
Other treatments	3	−1.01 [−1.86, −0.15]	0.02	83%	
Duration of disease					0.26
<10 y	5	−0.82 [−1.22, −0.42]	0.0004	81%	
≥10 y	1	−1.60 [−2.91, −0.29]	0.02	NA	
**TC**					
All comparisons	3	−0.50 [−0.61, −0.39]	<0.00001	0%	
Age					0.20
<50 y	1	−1.02 [−1.83, −0.21]	0.01	NA	
≥50 y	2	−0.49 [−0.60, −0.38]	<0.00001	0%	
Course of treatment					0.97
<8 w	1	−0.50 [−0.68, −0.32]	<0.00001	NA	
≥8 w	2	−0.50 [−0.64, −0.36]	<0.00001	40%	
**CRP**					
All comparisons	4	−0.58 [−0.88, −0.28]	0.0005	83%	
Age					0.84
<50 y	1	−0.55 [−0.69, −0.41]	<0.00001	NA	
≥50 y	3	−0.61 [−1.18, −0.05]	0.03	88%	
Safety					0.05
No adverse effects	1	−1.13 [−1.49, −0.77]	<0.00001	NA	
Adverse effects occurring	3	−0.62 [−0.97, −0.26]	0.0007	89%	
Region					0.81
Henan province	2	−0.81 [−1.38, −0.25]	0.005	88%	
Other provinces	2	−0.69 [−1.52, 0.14]	0.10	94%	
**Overall effective rate**		RR (95%CI)			
All comparisons	6	1.21 [1.12, 1.31]	<0.00001	0%	
Age					0.90
<50 y	2	1.22 [1.08, 1.38]	0.001	0%	
≥50 y	4	1.21 [1.09, 1.34]	0.0002	0%	
Different control treatment					0.79
Metformin	3	1.20 [1.08, 1.34]	0.001	0%	
Other treatments	3	1.23 [1.10, 1.37]	0.0003	0%	
Course of treatment					0.27
<8 w	1	1.10 [0.92, 1.33]	0.30	NA	
≥8 w	5	1.24 [1.14, 1.35]	<0.00001	0%	
Region					0.50
Henan province	2	1.18 [1.06, 1.30]	0.002	0%	
Other provinces	4	1.24 [1.11, 1.38]	0.0001	0%	

FBG, fasting blood glucose; 2hPG, two-hour postprandial glucose; HbA1c, glycosylated hemoglobin; TC, total cholesterol; CRP, c-reactive protein; Y, year; W, week.

## Abbreviations

2hPG: 2-hour postprandial glucose
CFDA: China Food and Drug Administration
CIs: Confidence intervals
CNKI: China National Knowledge Infrastructure
CQVIP: VIP information resource integration service platform
CRP: c-Reactive protein
GRADE: Grading of recommendations assessment, development, and evaluation
HbA1c: Glycated hemoglobin
FBG: Fasting blood glucose
OGTT: Oral glucose tolerance test
PRISMA-P: Preferred reporting items for systematic review and meta-analysis protocols
RCTs: Randomized controlled trials
T2DM: Type 2 diabetes mellitus
TC: Total cholesterol
RR: Risk ratio
TCM: Traditional Chinese medicine
VEGF: Vascular endothelial growth factor
WMDs: Weighted mean differences.
YQP: Yuquan Pill.

## Supplementary Materials

Supplementary Materials Supplementary Material 1: Subgroup analysis for FBG (age). Supplementary Material 2: Subgroup analysis for FBG (different control treatment). Supplementary Material 3: Subgroup analysis for 2hPG (age). Supplementary Material 4: Subgroup analysis for 2hPG (different control treatment). Supplementary Material 5: Subgroup analysis for 2hPG (course of treatment). Supplementary Material 6: Subgroup analysis for HbA1c (age). Supplementary Material 7: Subgroup analysis for HbA1c (different control treatment). Supplementary Material 8: Subgroup analysis for HbA1c (duration of disease). Supplementary Material 9: Subgroup analysis for TC (age). Supplementary Material 10: Subgroup analysis for TC (course of treatment). Supplementary Material 11: Subgroup analysis for CRP (age). Supplementary Material 12: Subgroup analysis for CRP (safety). Supplementary Material 13: Subgroup analysis for CRP (region). Supplementary Material 14: Subgroup analyses for overall effective rate (age). Supplementary Material 15: Subgroup analyses for overall effective rate (different control treatment). Supplementary Material 16: Subgroup analyses for overall effective rate (course of treatment). Supplementary Material 17: Subgroup analyses for overall effective rate (region).
