# Supplementary figures and images for: Efficacy and Safety of the Chinese Patent Medicine Yuquan Pill on Type 2 Diabetes Mellitus Patients: A Systematic Review and Meta-Analysis
Source: Evid Based Complement Alternat Med. 2021 Dec 2;2021:2562590. doi: 10.1155/2021/2562590 (PMC8660199; doi:10.1155/2021/2562590)

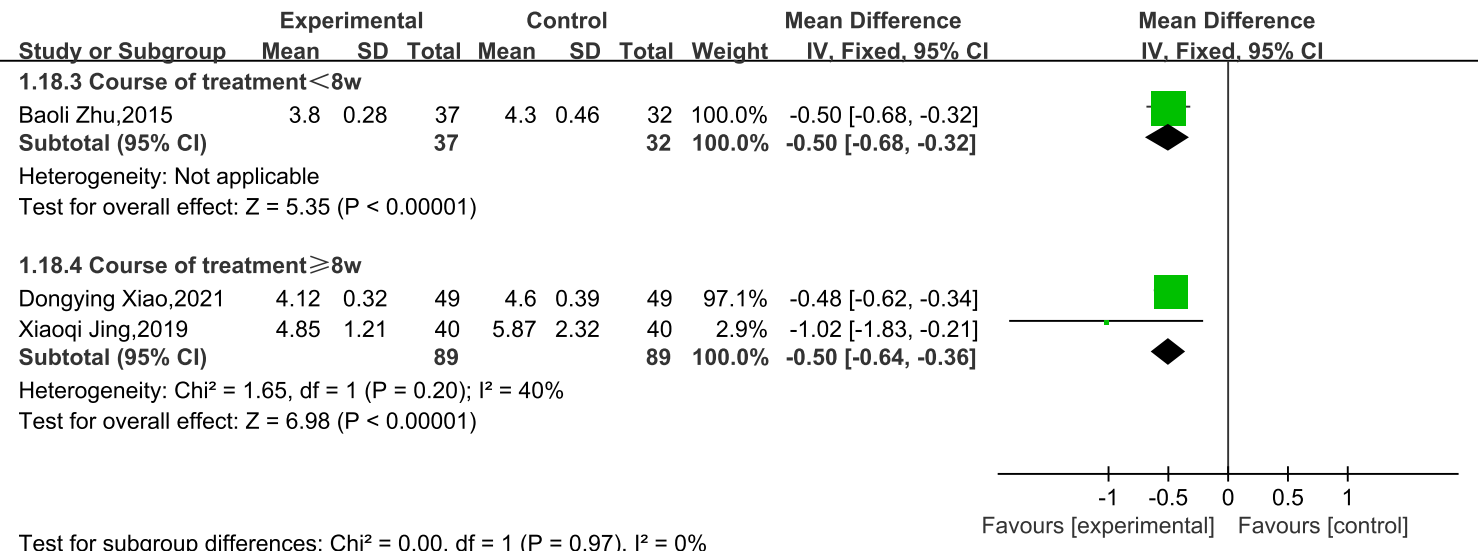

Supplement: Supplementary Materials — Supplementary Material 1: Subgroup analysis for FBG (age). Supplementary Material 2: Subgroup analysis for FBG (different control treatment). Supplementary Material 3: Subgroup analysis for 2hPG (age). Supplementary Material 4: Subgroup analysis for 2hPG (different control treatment). Supplementary Material 5: Subgroup analysis for 2hPG (course of treatment). Supplementary Material 6: Subgroup analysis for HbA1c (age). Supplementary Material 7: Subgroup analysis for HbA1c (different control treatment). Supplementary Material 8: Subgroup analysis for HbA1c (duration of disease). Supplementary Material 9: Subgroup analysis for TC (age). Supplementary Material 10: Subgroup analysis for TC (course of treatment). Supplementary Material 11: Subgroup analysis for CRP (age). Supplementary Material 12: Subgroup analysis for CRP (safety). Supplementary Material 13: Subgroup analysis for CRP (region). Supplementary Material 14: Subgroup analyses for overall effective rate (age). Supplementary Material 15: Subgroup analyses for overall effective rate (different control treatment). Supplementary Material 16: Subgroup analyses for overall effective rate (course of treatment). Supplementary Material 17: Subgroup analyses for overall effective rate (region). [file 2562590.f1.zip › 2562590.f1/Supplementary material10-Subgroup analysis for TC (Course of treatment).pdf]

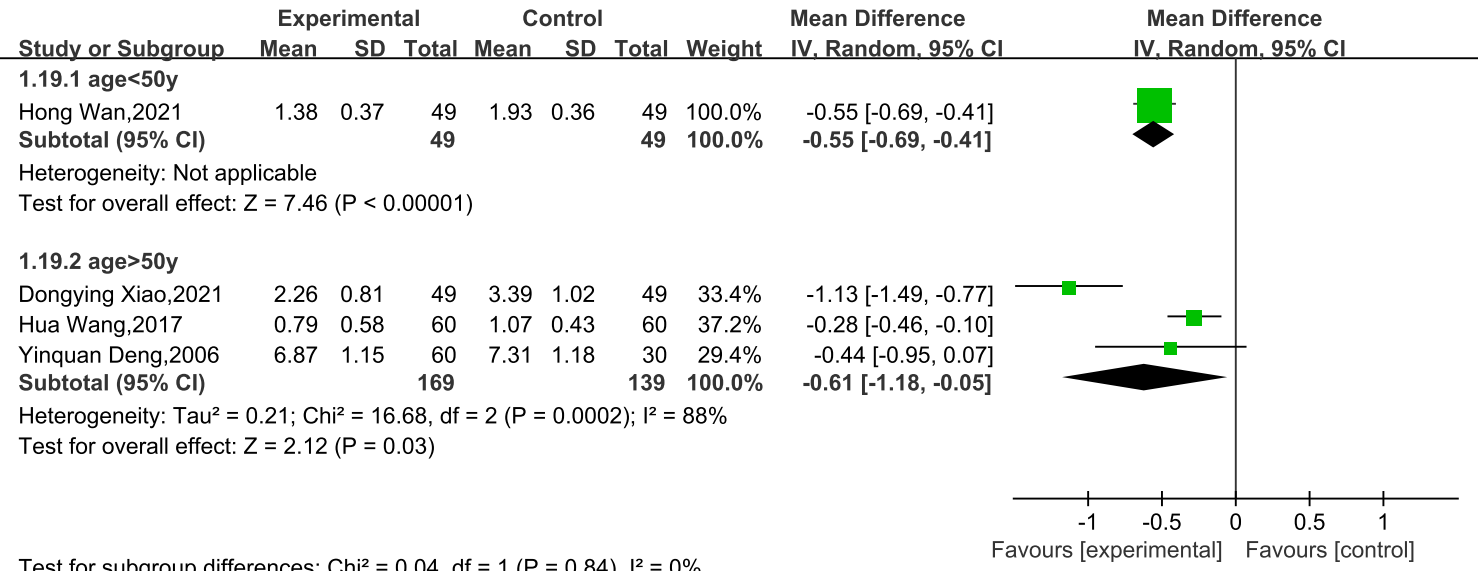

Supplement: Supplementary Materials — Supplementary Material 1: Subgroup analysis for FBG (age). Supplementary Material 2: Subgroup analysis for FBG (different control treatment). Supplementary Material 3: Subgroup analysis for 2hPG (age). Supplementary Material 4: Subgroup analysis for 2hPG (different control treatment). Supplementary Material 5: Subgroup analysis for 2hPG (course of treatment). Supplementary Material 6: Subgroup analysis for HbA1c (age). Supplementary Material 7: Subgroup analysis for HbA1c (different control treatment). Supplementary Material 8: Subgroup analysis for HbA1c (duration of disease). Supplementary Material 9: Subgroup analysis for TC (age). Supplementary Material 10: Subgroup analysis for TC (course of treatment). Supplementary Material 11: Subgroup analysis for CRP (age). Supplementary Material 12: Subgroup analysis for CRP (safety). Supplementary Material 13: Subgroup analysis for CRP (region). Supplementary Material 14: Subgroup analyses for overall effective rate (age). Supplementary Material 15: Subgroup analyses for overall effective rate (different control treatment). Supplementary Material 16: Subgroup analyses for overall effective rate (course of treatment). Supplementary Material 17: Subgroup analyses for overall effective rate (region). [file 2562590.f1.zip › 2562590.f1/Supplementary material11-Subgroup analysis for CRP(Age).pdf]

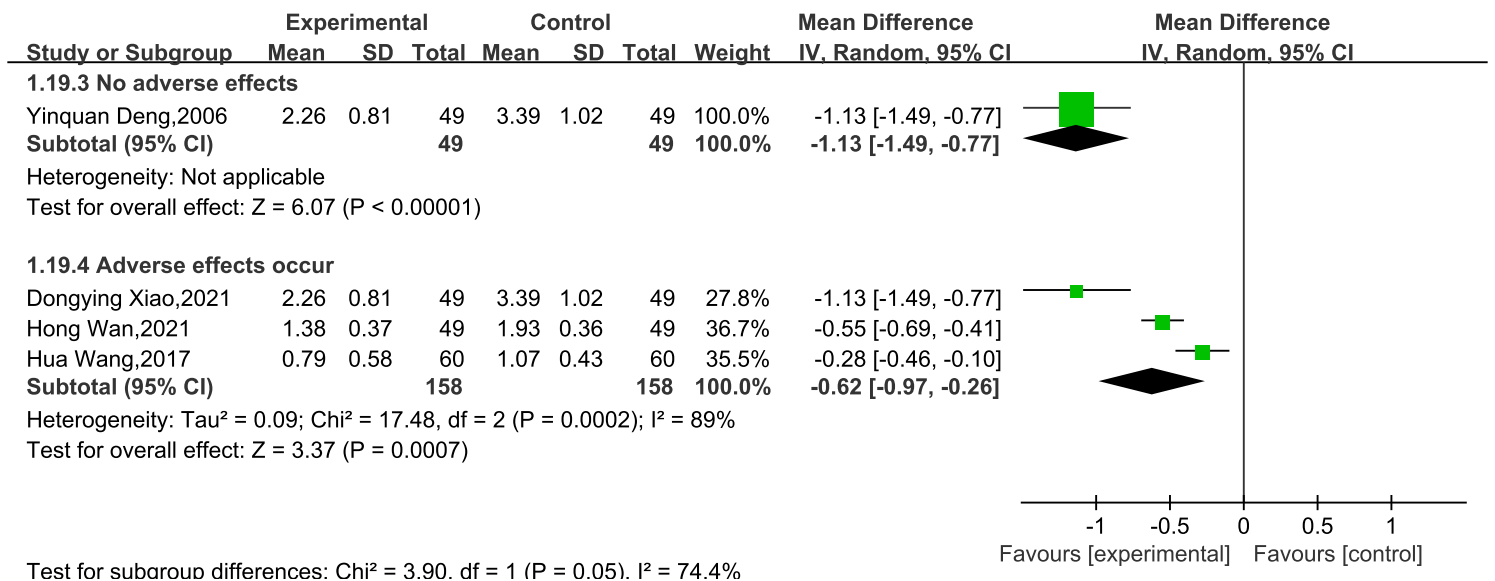

Supplement: Supplementary Materials — Supplementary Material 1: Subgroup analysis for FBG (age). Supplementary Material 2: Subgroup analysis for FBG (different control treatment). Supplementary Material 3: Subgroup analysis for 2hPG (age). Supplementary Material 4: Subgroup analysis for 2hPG (different control treatment). Supplementary Material 5: Subgroup analysis for 2hPG (course of treatment). Supplementary Material 6: Subgroup analysis for HbA1c (age). Supplementary Material 7: Subgroup analysis for HbA1c (different control treatment). Supplementary Material 8: Subgroup analysis for HbA1c (duration of disease). Supplementary Material 9: Subgroup analysis for TC (age). Supplementary Material 10: Subgroup analysis for TC (course of treatment). Supplementary Material 11: Subgroup analysis for CRP (age). Supplementary Material 12: Subgroup analysis for CRP (safety). Supplementary Material 13: Subgroup analysis for CRP (region). Supplementary Material 14: Subgroup analyses for overall effective rate (age). Supplementary Material 15: Subgroup analyses for overall effective rate (different control treatment). Supplementary Material 16: Subgroup analyses for overall effective rate (course of treatment). Supplementary Material 17: Subgroup analyses for overall effective rate (region). [file 2562590.f1.zip › 2562590.f1/Supplementary material12-Subgroup analysis for CRP (Safety).pdf]

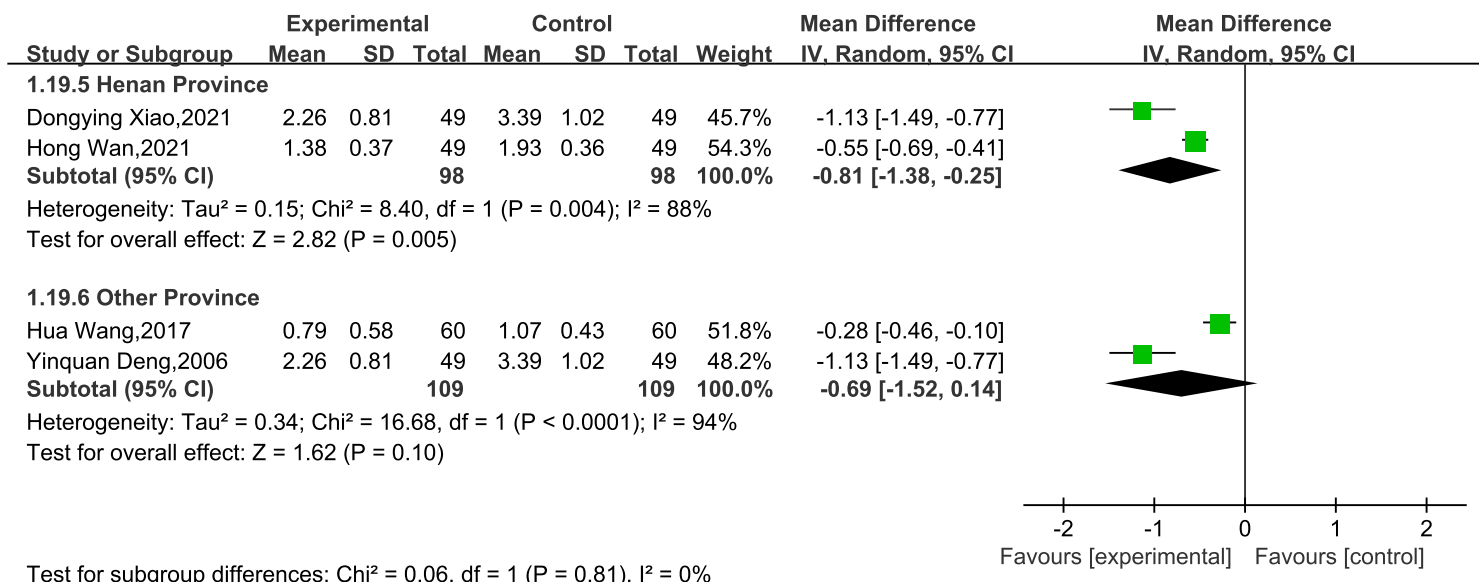

Supplement: Supplementary Materials — Supplementary Material 1: Subgroup analysis for FBG (age). Supplementary Material 2: Subgroup analysis for FBG (different control treatment). Supplementary Material 3: Subgroup analysis for 2hPG (age). Supplementary Material 4: Subgroup analysis for 2hPG (different control treatment). Supplementary Material 5: Subgroup analysis for 2hPG (course of treatment). Supplementary Material 6: Subgroup analysis for HbA1c (age). Supplementary Material 7: Subgroup analysis for HbA1c (different control treatment). Supplementary Material 8: Subgroup analysis for HbA1c (duration of disease). Supplementary Material 9: Subgroup analysis for TC (age). Supplementary Material 10: Subgroup analysis for TC (course of treatment). Supplementary Material 11: Subgroup analysis for CRP (age). Supplementary Material 12: Subgroup analysis for CRP (safety). Supplementary Material 13: Subgroup analysis for CRP (region). Supplementary Material 14: Subgroup analyses for overall effective rate (age). Supplementary Material 15: Subgroup analyses for overall effective rate (different control treatment). Supplementary Material 16: Subgroup analyses for overall effective rate (course of treatment). Supplementary Material 17: Subgroup analyses for overall effective rate (region). [file 2562590.f1.zip › 2562590.f1/Supplementary material13-Subgroup analysis for CRP (Region).pdf]

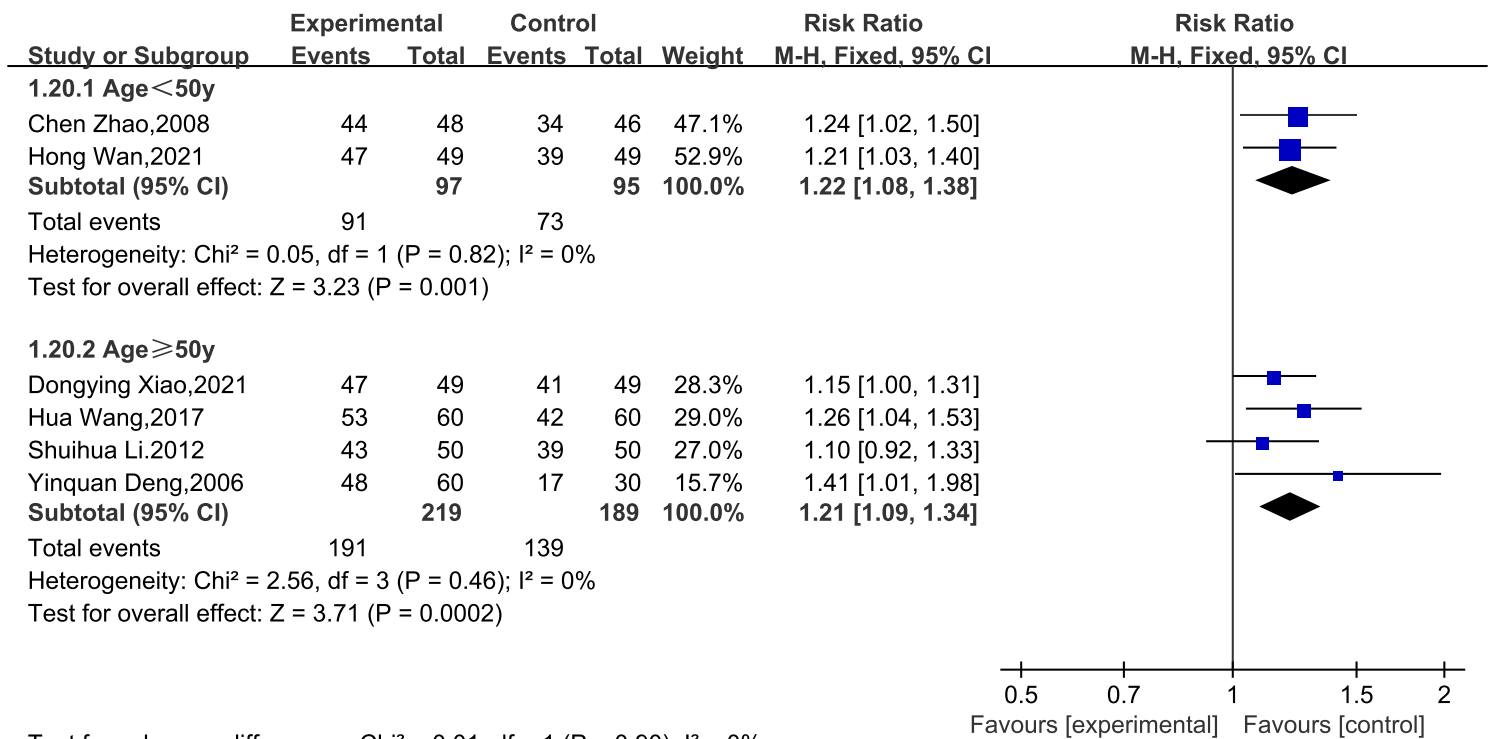

Supplement: Supplementary Materials — Supplementary Material 1: Subgroup analysis for FBG (age). Supplementary Material 2: Subgroup analysis for FBG (different control treatment). Supplementary Material 3: Subgroup analysis for 2hPG (age). Supplementary Material 4: Subgroup analysis for 2hPG (different control treatment). Supplementary Material 5: Subgroup analysis for 2hPG (course of treatment). Supplementary Material 6: Subgroup analysis for HbA1c (age). Supplementary Material 7: Subgroup analysis for HbA1c (different control treatment). Supplementary Material 8: Subgroup analysis for HbA1c (duration of disease). Supplementary Material 9: Subgroup analysis for TC (age). Supplementary Material 10: Subgroup analysis for TC (course of treatment). Supplementary Material 11: Subgroup analysis for CRP (age). Supplementary Material 12: Subgroup analysis for CRP (safety). Supplementary Material 13: Subgroup analysis for CRP (region). Supplementary Material 14: Subgroup analyses for overall effective rate (age). Supplementary Material 15: Subgroup analyses for overall effective rate (different control treatment). Supplementary Material 16: Subgroup analyses for overall effective rate (course of treatment). Supplementary Material 17: Subgroup analyses for overall effective rate (region). [file 2562590.f1.zip › 2562590.f1/Supplementary material14-Subgroup analyses for Overall effective rate (Age).pdf]

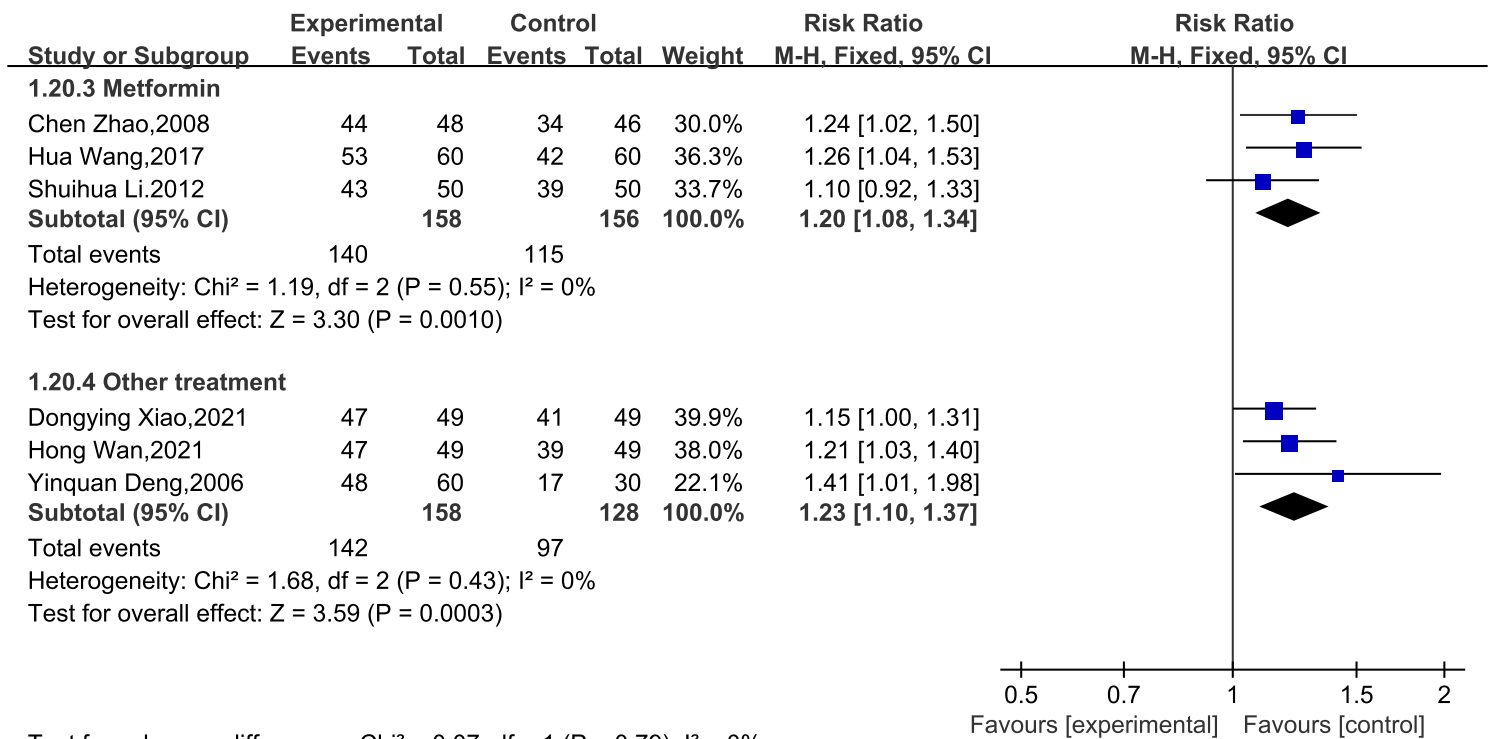

Supplement: Supplementary Materials — Supplementary Material 1: Subgroup analysis for FBG (age). Supplementary Material 2: Subgroup analysis for FBG (different control treatment). Supplementary Material 3: Subgroup analysis for 2hPG (age). Supplementary Material 4: Subgroup analysis for 2hPG (different control treatment). Supplementary Material 5: Subgroup analysis for 2hPG (course of treatment). Supplementary Material 6: Subgroup analysis for HbA1c (age). Supplementary Material 7: Subgroup analysis for HbA1c (different control treatment). Supplementary Material 8: Subgroup analysis for HbA1c (duration of disease). Supplementary Material 9: Subgroup analysis for TC (age). Supplementary Material 10: Subgroup analysis for TC (course of treatment). Supplementary Material 11: Subgroup analysis for CRP (age). Supplementary Material 12: Subgroup analysis for CRP (safety). Supplementary Material 13: Subgroup analysis for CRP (region). Supplementary Material 14: Subgroup analyses for overall effective rate (age). Supplementary Material 15: Subgroup analyses for overall effective rate (different control treatment). Supplementary Material 16: Subgroup analyses for overall effective rate (course of treatment). Supplementary Material 17: Subgroup analyses for overall effective rate (region). [file 2562590.f1.zip › 2562590.f1/Supplementary material15-Subgroup analyses for Overall effective rate (Different control treatment).pdf]

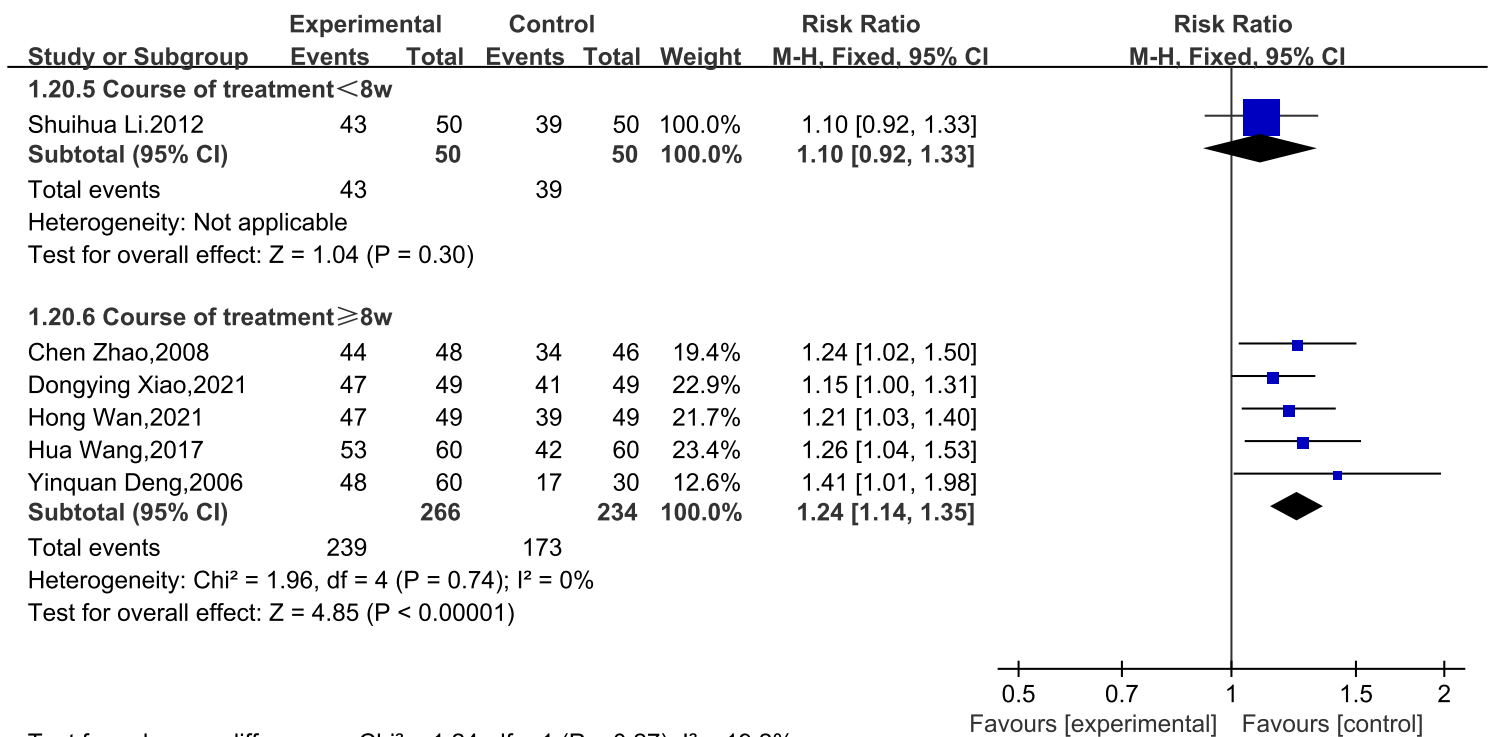

Supplement: Supplementary Materials — Supplementary Material 1: Subgroup analysis for FBG (age). Supplementary Material 2: Subgroup analysis for FBG (different control treatment). Supplementary Material 3: Subgroup analysis for 2hPG (age). Supplementary Material 4: Subgroup analysis for 2hPG (different control treatment). Supplementary Material 5: Subgroup analysis for 2hPG (course of treatment). Supplementary Material 6: Subgroup analysis for HbA1c (age). Supplementary Material 7: Subgroup analysis for HbA1c (different control treatment). Supplementary Material 8: Subgroup analysis for HbA1c (duration of disease). Supplementary Material 9: Subgroup analysis for TC (age). Supplementary Material 10: Subgroup analysis for TC (course of treatment). Supplementary Material 11: Subgroup analysis for CRP (age). Supplementary Material 12: Subgroup analysis for CRP (safety). Supplementary Material 13: Subgroup analysis for CRP (region). Supplementary Material 14: Subgroup analyses for overall effective rate (age). Supplementary Material 15: Subgroup analyses for overall effective rate (different control treatment). Supplementary Material 16: Subgroup analyses for overall effective rate (course of treatment). Supplementary Material 17: Subgroup analyses for overall effective rate (region). [file 2562590.f1.zip › 2562590.f1/Supplementary material16-Subgroup analyses for Overall effective rate (Course of treatment).pdf]

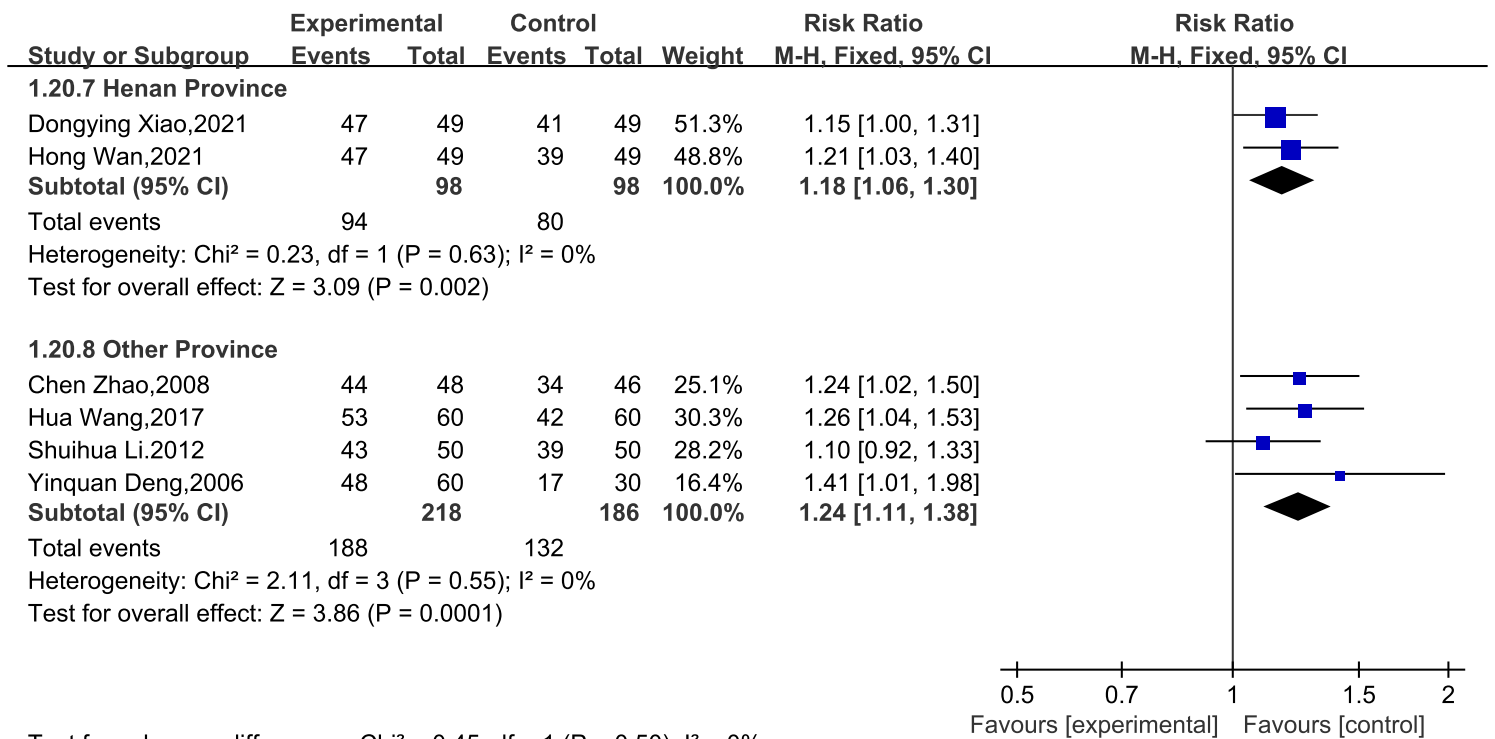

Supplement: Supplementary Materials — Supplementary Material 1: Subgroup analysis for FBG (age). Supplementary Material 2: Subgroup analysis for FBG (different control treatment). Supplementary Material 3: Subgroup analysis for 2hPG (age). Supplementary Material 4: Subgroup analysis for 2hPG (different control treatment). Supplementary Material 5: Subgroup analysis for 2hPG (course of treatment). Supplementary Material 6: Subgroup analysis for HbA1c (age). Supplementary Material 7: Subgroup analysis for HbA1c (different control treatment). Supplementary Material 8: Subgroup analysis for HbA1c (duration of disease). Supplementary Material 9: Subgroup analysis for TC (age). Supplementary Material 10: Subgroup analysis for TC (course of treatment). Supplementary Material 11: Subgroup analysis for CRP (age). Supplementary Material 12: Subgroup analysis for CRP (safety). Supplementary Material 13: Subgroup analysis for CRP (region). Supplementary Material 14: Subgroup analyses for overall effective rate (age). Supplementary Material 15: Subgroup analyses for overall effective rate (different control treatment). Supplementary Material 16: Subgroup analyses for overall effective rate (course of treatment). Supplementary Material 17: Subgroup analyses for overall effective rate (region). [file 2562590.f1.zip › 2562590.f1/Supplementary material17-Subgroup analyses for Overall effective rate (Region).pdf]

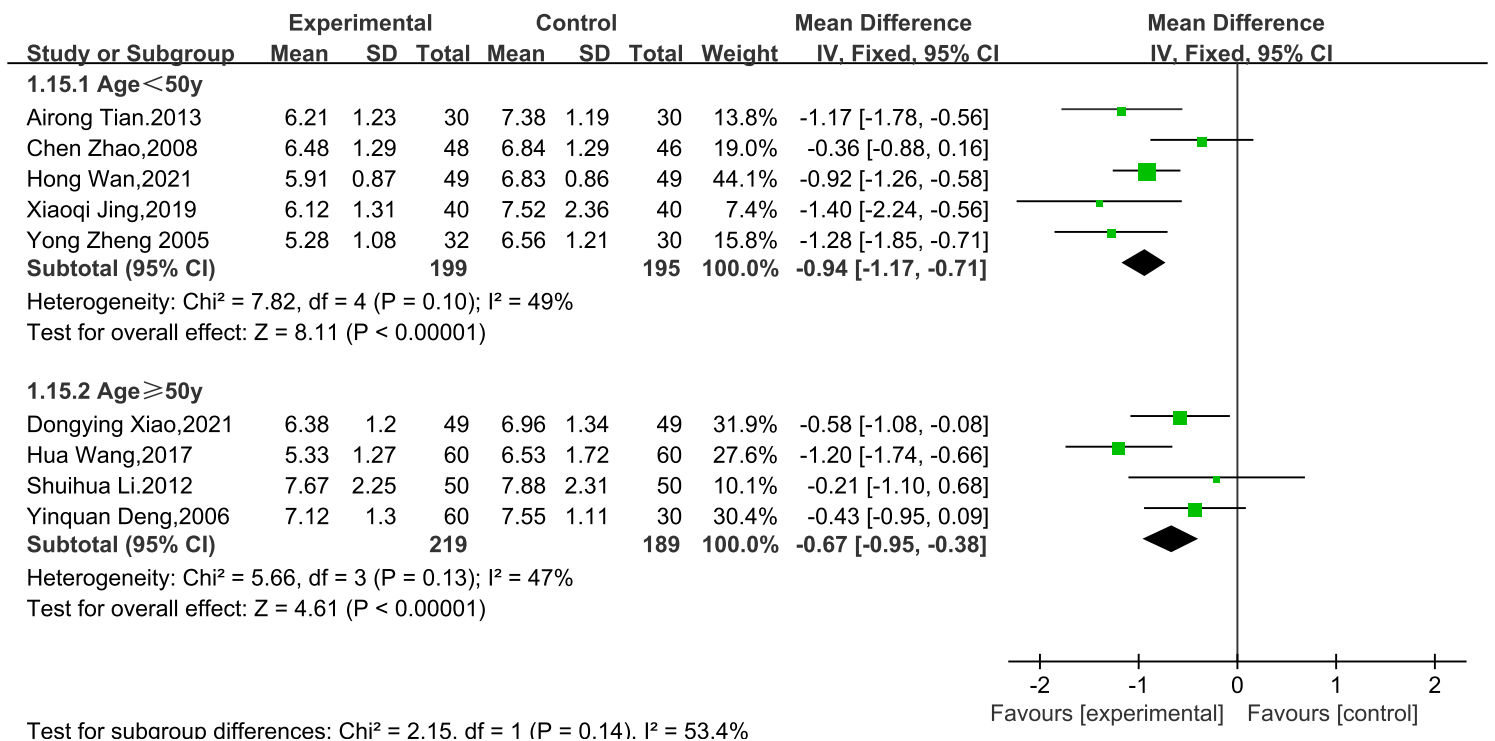

Supplement: Supplementary Materials — Supplementary Material 1: Subgroup analysis for FBG (age). Supplementary Material 2: Subgroup analysis for FBG (different control treatment). Supplementary Material 3: Subgroup analysis for 2hPG (age). Supplementary Material 4: Subgroup analysis for 2hPG (different control treatment). Supplementary Material 5: Subgroup analysis for 2hPG (course of treatment). Supplementary Material 6: Subgroup analysis for HbA1c (age). Supplementary Material 7: Subgroup analysis for HbA1c (different control treatment). Supplementary Material 8: Subgroup analysis for HbA1c (duration of disease). Supplementary Material 9: Subgroup analysis for TC (age). Supplementary Material 10: Subgroup analysis for TC (course of treatment). Supplementary Material 11: Subgroup analysis for CRP (age). Supplementary Material 12: Subgroup analysis for CRP (safety). Supplementary Material 13: Subgroup analysis for CRP (region). Supplementary Material 14: Subgroup analyses for overall effective rate (age). Supplementary Material 15: Subgroup analyses for overall effective rate (different control treatment). Supplementary Material 16: Subgroup analyses for overall effective rate (course of treatment). Supplementary Material 17: Subgroup analyses for overall effective rate (region). [file 2562590.f1.zip › 2562590.f1/Supplementary material1-Subgroup analysis for FBG (Age).pdf]

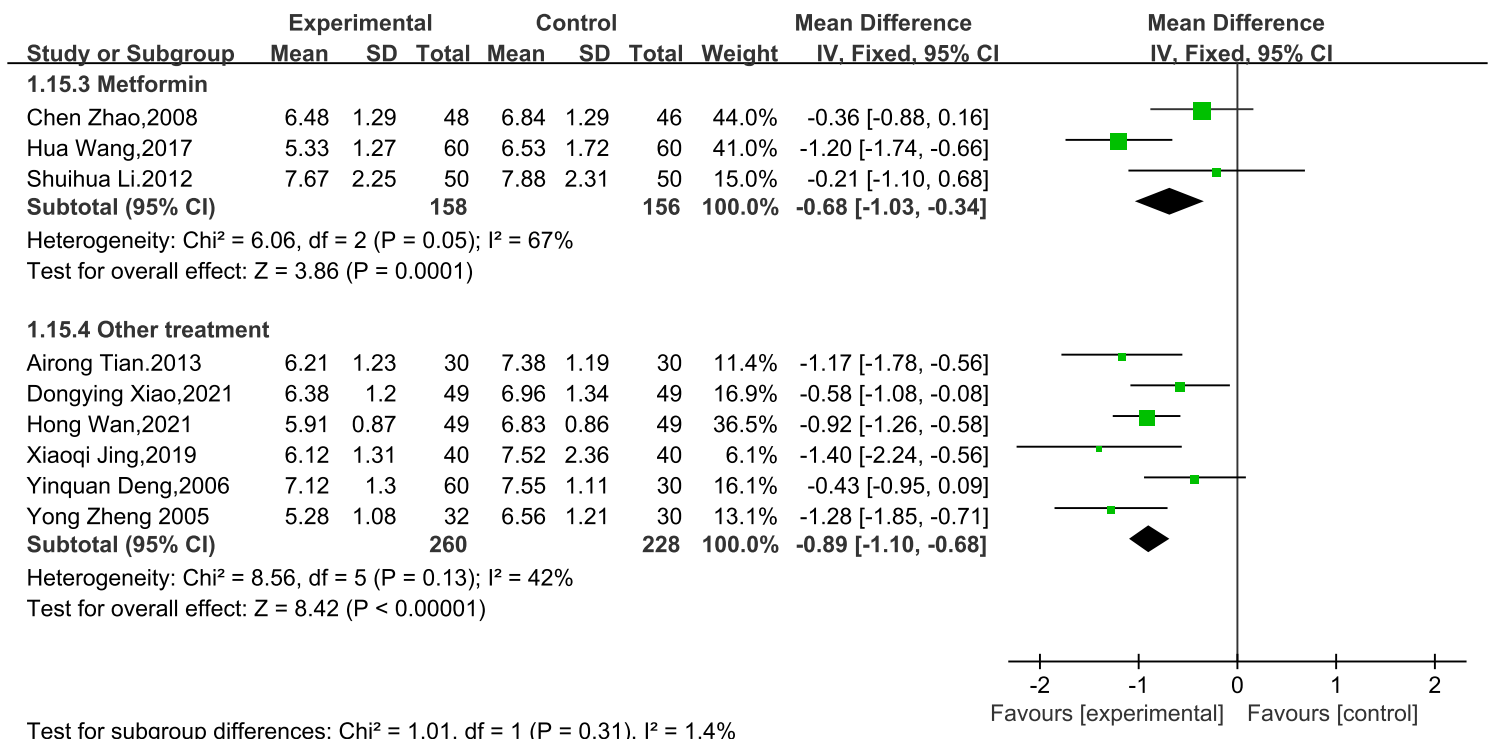

Supplement: Supplementary Materials — Supplementary Material 1: Subgroup analysis for FBG (age). Supplementary Material 2: Subgroup analysis for FBG (different control treatment). Supplementary Material 3: Subgroup analysis for 2hPG (age). Supplementary Material 4: Subgroup analysis for 2hPG (different control treatment). Supplementary Material 5: Subgroup analysis for 2hPG (course of treatment). Supplementary Material 6: Subgroup analysis for HbA1c (age). Supplementary Material 7: Subgroup analysis for HbA1c (different control treatment). Supplementary Material 8: Subgroup analysis for HbA1c (duration of disease). Supplementary Material 9: Subgroup analysis for TC (age). Supplementary Material 10: Subgroup analysis for TC (course of treatment). Supplementary Material 11: Subgroup analysis for CRP (age). Supplementary Material 12: Subgroup analysis for CRP (safety). Supplementary Material 13: Subgroup analysis for CRP (region). Supplementary Material 14: Subgroup analyses for overall effective rate (age). Supplementary Material 15: Subgroup analyses for overall effective rate (different control treatment). Supplementary Material 16: Subgroup analyses for overall effective rate (course of treatment). Supplementary Material 17: Subgroup analyses for overall effective rate (region). [file 2562590.f1.zip › 2562590.f1/Supplementary material2-Subgroup analysis for FBG (Different control treatment).pdf]

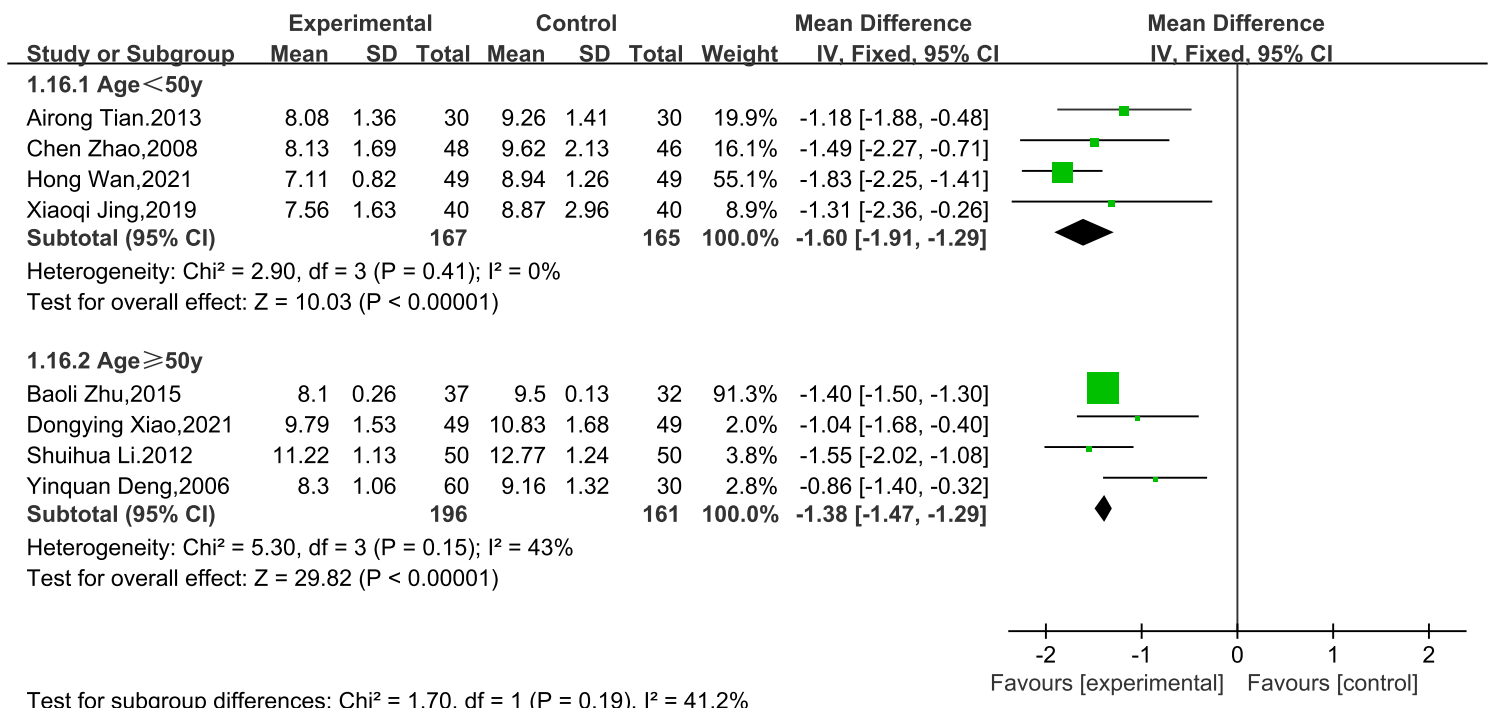

Supplement: Supplementary Materials — Supplementary Material 1: Subgroup analysis for FBG (age). Supplementary Material 2: Subgroup analysis for FBG (different control treatment). Supplementary Material 3: Subgroup analysis for 2hPG (age). Supplementary Material 4: Subgroup analysis for 2hPG (different control treatment). Supplementary Material 5: Subgroup analysis for 2hPG (course of treatment). Supplementary Material 6: Subgroup analysis for HbA1c (age). Supplementary Material 7: Subgroup analysis for HbA1c (different control treatment). Supplementary Material 8: Subgroup analysis for HbA1c (duration of disease). Supplementary Material 9: Subgroup analysis for TC (age). Supplementary Material 10: Subgroup analysis for TC (course of treatment). Supplementary Material 11: Subgroup analysis for CRP (age). Supplementary Material 12: Subgroup analysis for CRP (safety). Supplementary Material 13: Subgroup analysis for CRP (region). Supplementary Material 14: Subgroup analyses for overall effective rate (age). Supplementary Material 15: Subgroup analyses for overall effective rate (different control treatment). Supplementary Material 16: Subgroup analyses for overall effective rate (course of treatment). Supplementary Material 17: Subgroup analyses for overall effective rate (region). [file 2562590.f1.zip › 2562590.f1/Supplementary material3-Subgroup analysis for 2hPG (Age).pdf]

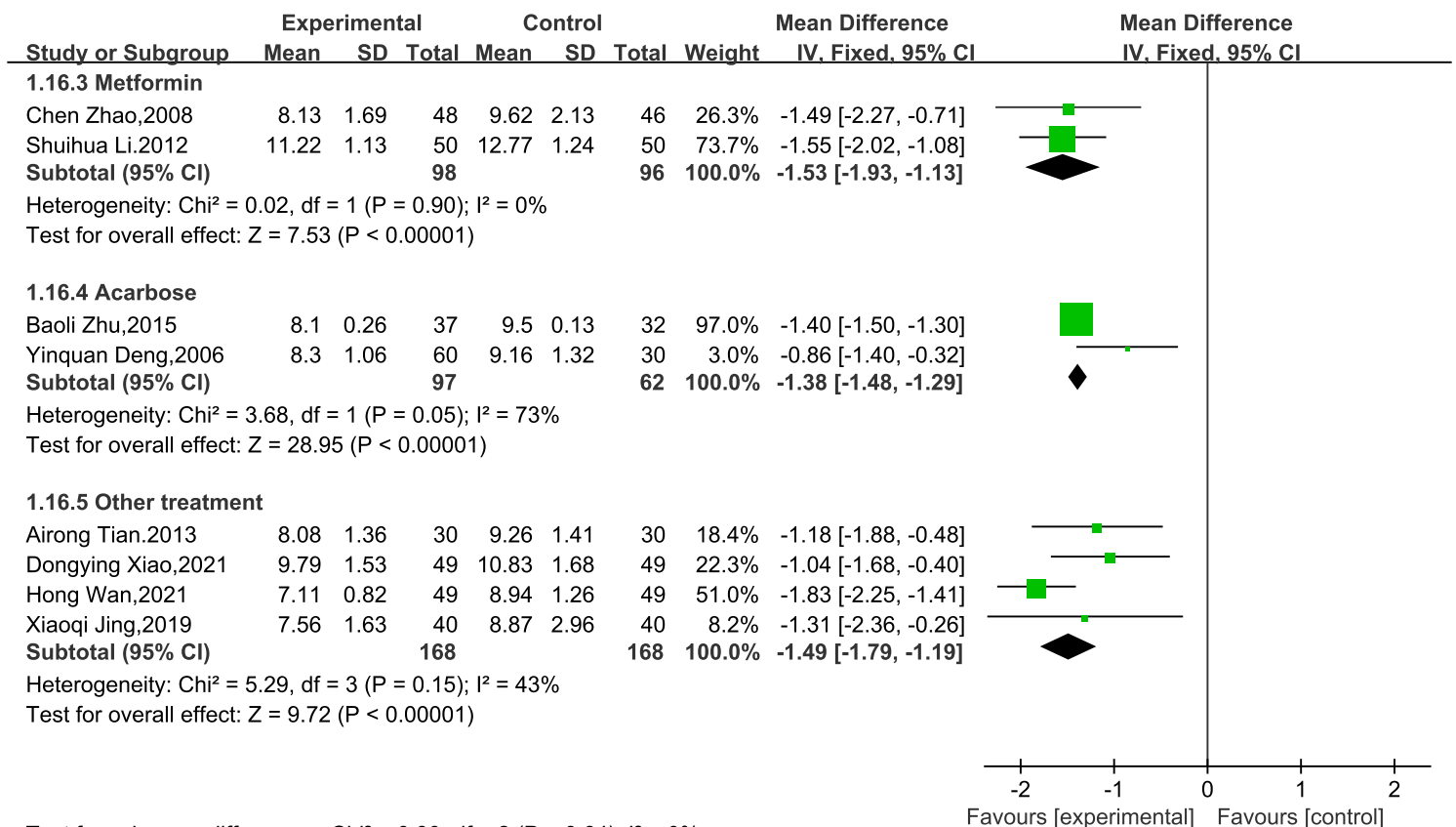

Test for subaroup differences:  $\text{Chi}^2 = 0.90$ ,  $\text{df} = 2$  ( $P = 0.64$ ).  $I^2 = 0\%$

Supplement: Supplementary Materials — Supplementary Material 1: Subgroup analysis for FBG (age). Supplementary Material 2: Subgroup analysis for FBG (different control treatment). Supplementary Material 3: Subgroup analysis for 2hPG (age). Supplementary Material 4: Subgroup analysis for 2hPG (different control treatment). Supplementary Material 5: Subgroup analysis for 2hPG (course of treatment). Supplementary Material 6: Subgroup analysis for HbA1c (age). Supplementary Material 7: Subgroup analysis for HbA1c (different control treatment). Supplementary Material 8: Subgroup analysis for HbA1c (duration of disease). Supplementary Material 9: Subgroup analysis for TC (age). Supplementary Material 10: Subgroup analysis for TC (course of treatment). Supplementary Material 11: Subgroup analysis for CRP (age). Supplementary Material 12: Subgroup analysis for CRP (safety). Supplementary Material 13: Subgroup analysis for CRP (region). Supplementary Material 14: Subgroup analyses for overall effective rate (age). Supplementary Material 15: Subgroup analyses for overall effective rate (different control treatment). Supplementary Material 16: Subgroup analyses for overall effective rate (course of treatment). Supplementary Material 17: Subgroup analyses for overall effective rate (region). [file 2562590.f1.zip › 2562590.f1/Supplementary material4-Subgroup analysis for 2hPG (Different control treatment).pdf]

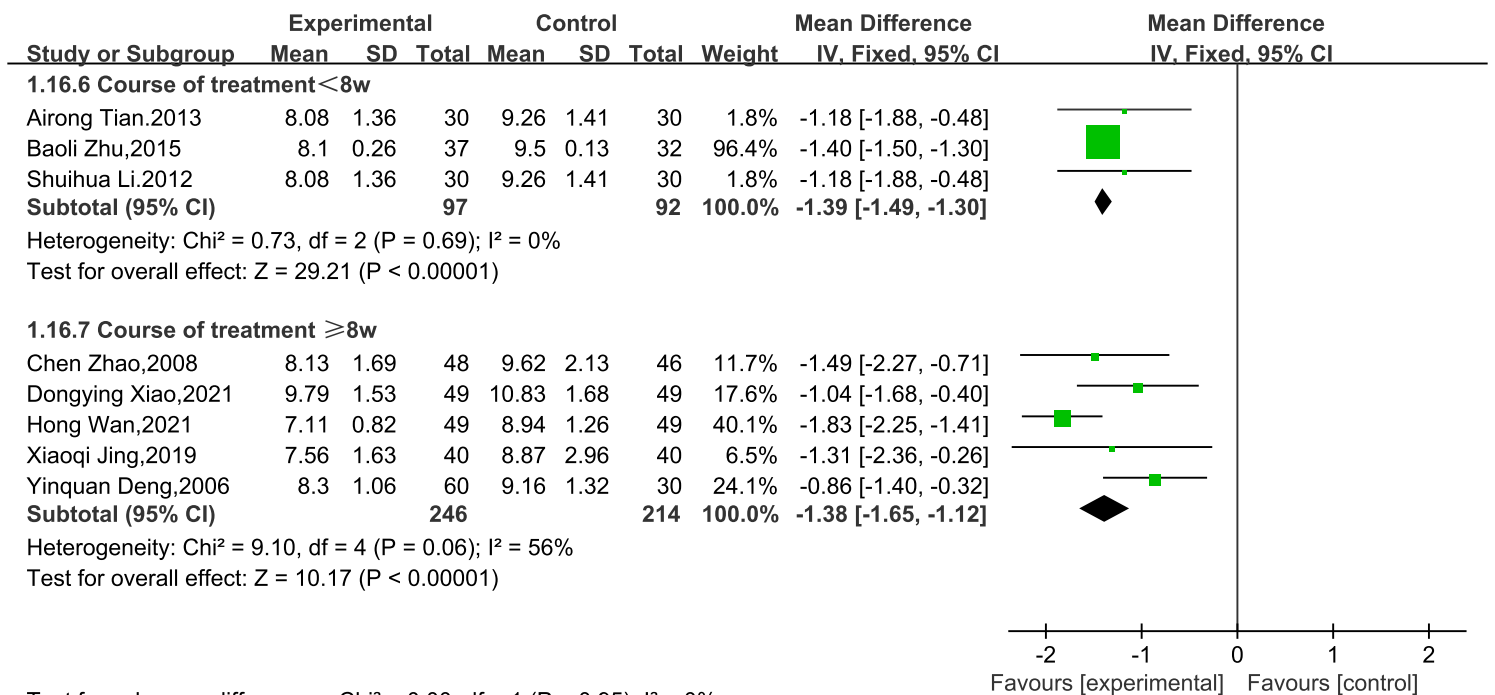

Supplement: Supplementary Materials — Supplementary Material 1: Subgroup analysis for FBG (age). Supplementary Material 2: Subgroup analysis for FBG (different control treatment). Supplementary Material 3: Subgroup analysis for 2hPG (age). Supplementary Material 4: Subgroup analysis for 2hPG (different control treatment). Supplementary Material 5: Subgroup analysis for 2hPG (course of treatment). Supplementary Material 6: Subgroup analysis for HbA1c (age). Supplementary Material 7: Subgroup analysis for HbA1c (different control treatment). Supplementary Material 8: Subgroup analysis for HbA1c (duration of disease). Supplementary Material 9: Subgroup analysis for TC (age). Supplementary Material 10: Subgroup analysis for TC (course of treatment). Supplementary Material 11: Subgroup analysis for CRP (age). Supplementary Material 12: Subgroup analysis for CRP (safety). Supplementary Material 13: Subgroup analysis for CRP (region). Supplementary Material 14: Subgroup analyses for overall effective rate (age). Supplementary Material 15: Subgroup analyses for overall effective rate (different control treatment). Supplementary Material 16: Subgroup analyses for overall effective rate (course of treatment). Supplementary Material 17: Subgroup analyses for overall effective rate (region). [file 2562590.f1.zip › 2562590.f1/Supplementary material5-Subgroup analysis for 2hPG (Course of treatment).pdf]

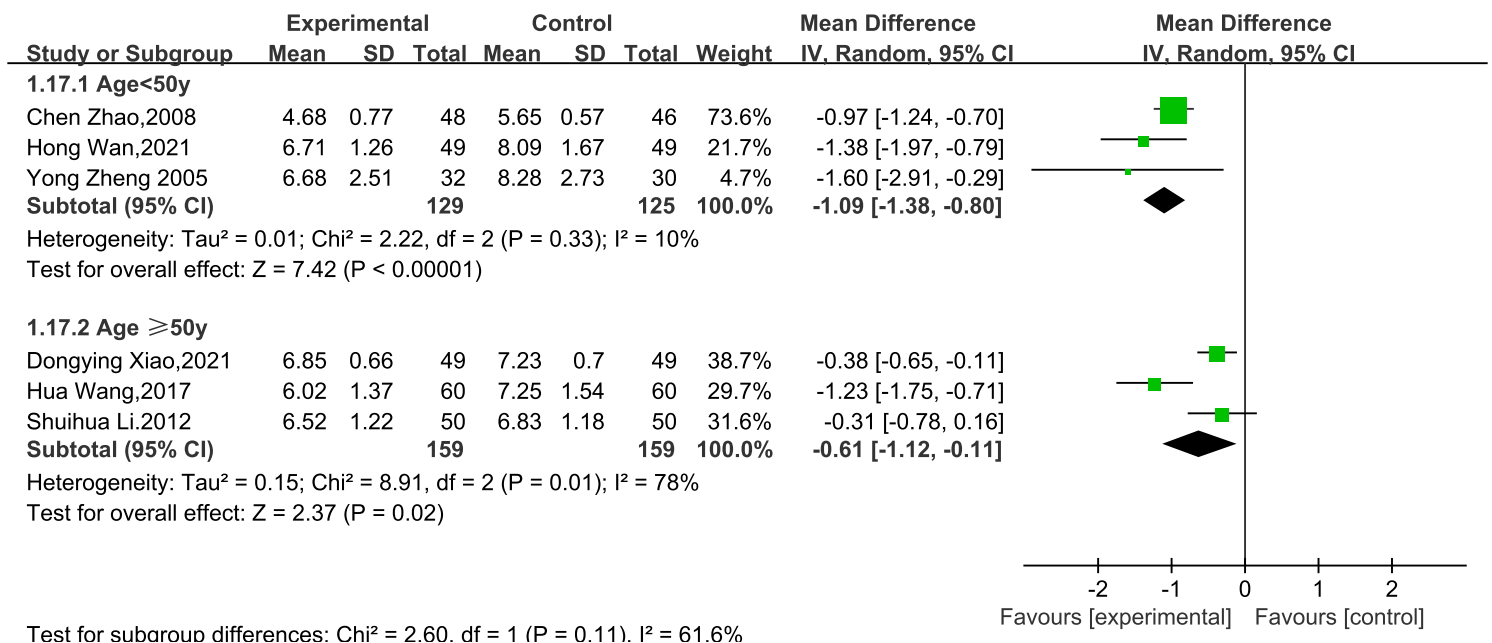

Supplement: Supplementary Materials — Supplementary Material 1: Subgroup analysis for FBG (age). Supplementary Material 2: Subgroup analysis for FBG (different control treatment). Supplementary Material 3: Subgroup analysis for 2hPG (age). Supplementary Material 4: Subgroup analysis for 2hPG (different control treatment). Supplementary Material 5: Subgroup analysis for 2hPG (course of treatment). Supplementary Material 6: Subgroup analysis for HbA1c (age). Supplementary Material 7: Subgroup analysis for HbA1c (different control treatment). Supplementary Material 8: Subgroup analysis for HbA1c (duration of disease). Supplementary Material 9: Subgroup analysis for TC (age). Supplementary Material 10: Subgroup analysis for TC (course of treatment). Supplementary Material 11: Subgroup analysis for CRP (age). Supplementary Material 12: Subgroup analysis for CRP (safety). Supplementary Material 13: Subgroup analysis for CRP (region). Supplementary Material 14: Subgroup analyses for overall effective rate (age). Supplementary Material 15: Subgroup analyses for overall effective rate (different control treatment). Supplementary Material 16: Subgroup analyses for overall effective rate (course of treatment). Supplementary Material 17: Subgroup analyses for overall effective rate (region). [file 2562590.f1.zip › 2562590.f1/Supplementary material6-Subgroup analysis for HbA1c (Age).pdf]

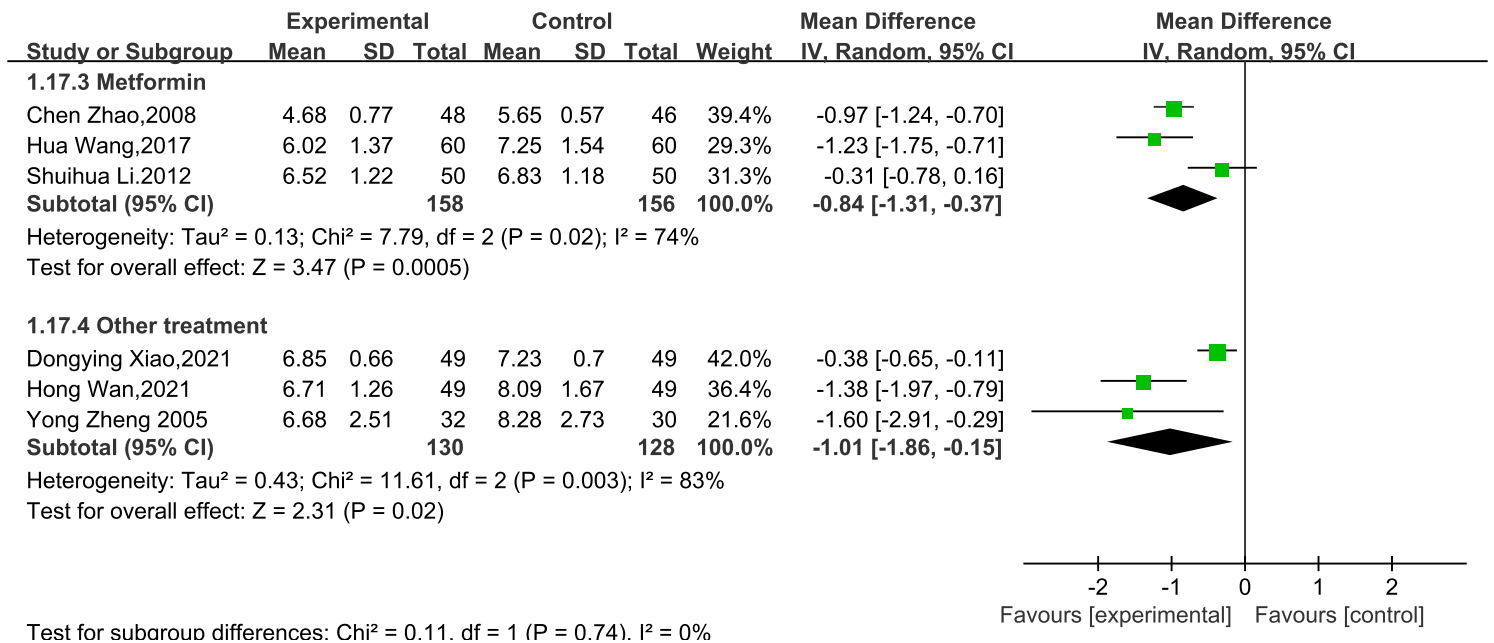

Supplement: Supplementary Materials — Supplementary Material 1: Subgroup analysis for FBG (age). Supplementary Material 2: Subgroup analysis for FBG (different control treatment). Supplementary Material 3: Subgroup analysis for 2hPG (age). Supplementary Material 4: Subgroup analysis for 2hPG (different control treatment). Supplementary Material 5: Subgroup analysis for 2hPG (course of treatment). Supplementary Material 6: Subgroup analysis for HbA1c (age). Supplementary Material 7: Subgroup analysis for HbA1c (different control treatment). Supplementary Material 8: Subgroup analysis for HbA1c (duration of disease). Supplementary Material 9: Subgroup analysis for TC (age). Supplementary Material 10: Subgroup analysis for TC (course of treatment). Supplementary Material 11: Subgroup analysis for CRP (age). Supplementary Material 12: Subgroup analysis for CRP (safety). Supplementary Material 13: Subgroup analysis for CRP (region). Supplementary Material 14: Subgroup analyses for overall effective rate (age). Supplementary Material 15: Subgroup analyses for overall effective rate (different control treatment). Supplementary Material 16: Subgroup analyses for overall effective rate (course of treatment). Supplementary Material 17: Subgroup analyses for overall effective rate (region). [file 2562590.f1.zip › 2562590.f1/Supplementary material7-Subgroup analysis for HbA1c (Different control treatment).pdf]

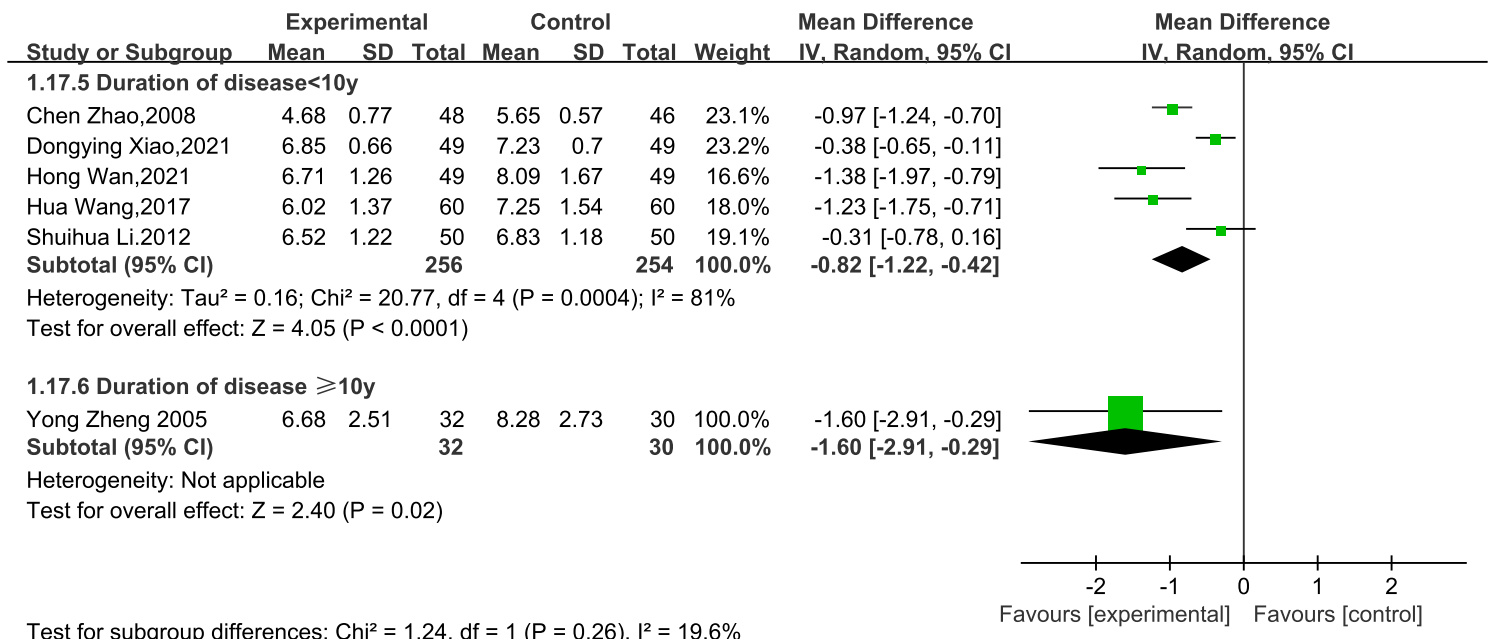

Supplement: Supplementary Materials — Supplementary Material 1: Subgroup analysis for FBG (age). Supplementary Material 2: Subgroup analysis for FBG (different control treatment). Supplementary Material 3: Subgroup analysis for 2hPG (age). Supplementary Material 4: Subgroup analysis for 2hPG (different control treatment). Supplementary Material 5: Subgroup analysis for 2hPG (course of treatment). Supplementary Material 6: Subgroup analysis for HbA1c (age). Supplementary Material 7: Subgroup analysis for HbA1c (different control treatment). Supplementary Material 8: Subgroup analysis for HbA1c (duration of disease). Supplementary Material 9: Subgroup analysis for TC (age). Supplementary Material 10: Subgroup analysis for TC (course of treatment). Supplementary Material 11: Subgroup analysis for CRP (age). Supplementary Material 12: Subgroup analysis for CRP (safety). Supplementary Material 13: Subgroup analysis for CRP (region). Supplementary Material 14: Subgroup analyses for overall effective rate (age). Supplementary Material 15: Subgroup analyses for overall effective rate (different control treatment). Supplementary Material 16: Subgroup analyses for overall effective rate (course of treatment). Supplementary Material 17: Subgroup analyses for overall effective rate (region). [file 2562590.f1.zip › 2562590.f1/Supplementary material8-Subgroup analysis for HbA1c (Duration of disease).pdf]

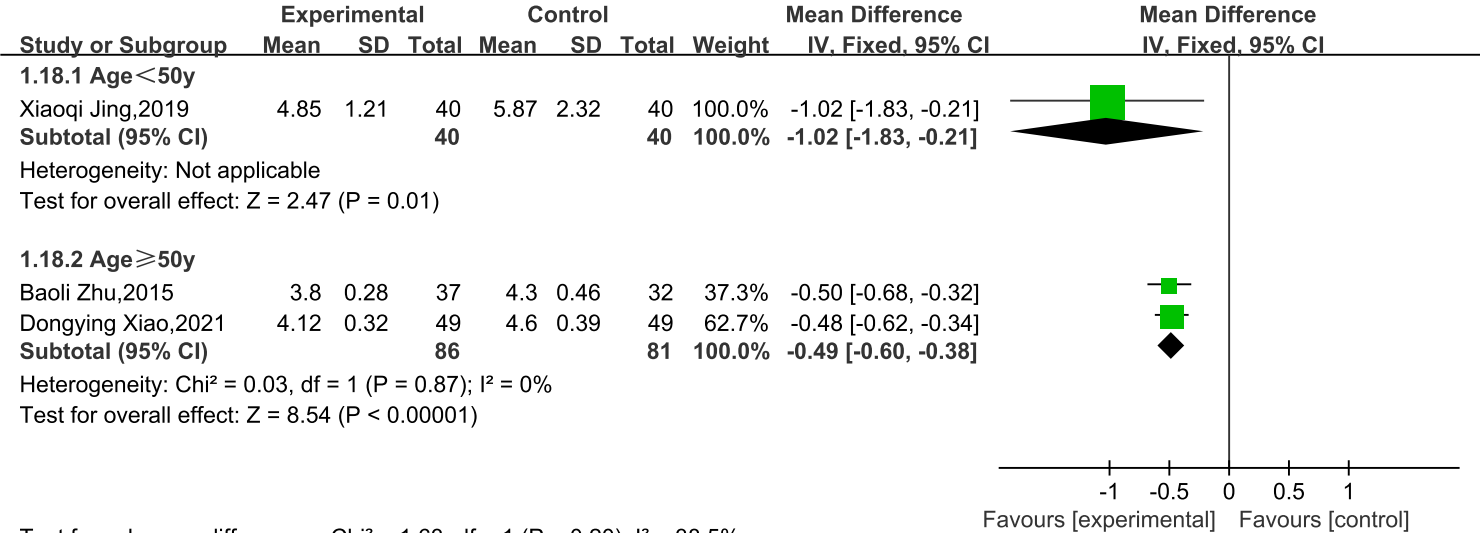

Supplement: Supplementary Materials — Supplementary Material 1: Subgroup analysis for FBG (age). Supplementary Material 2: Subgroup analysis for FBG (different control treatment). Supplementary Material 3: Subgroup analysis for 2hPG (age). Supplementary Material 4: Subgroup analysis for 2hPG (different control treatment). Supplementary Material 5: Subgroup analysis for 2hPG (course of treatment). Supplementary Material 6: Subgroup analysis for HbA1c (age). Supplementary Material 7: Subgroup analysis for HbA1c (different control treatment). Supplementary Material 8: Subgroup analysis for HbA1c (duration of disease). Supplementary Material 9: Subgroup analysis for TC (age). Supplementary Material 10: Subgroup analysis for TC (course of treatment). Supplementary Material 11: Subgroup analysis for CRP (age). Supplementary Material 12: Subgroup analysis for CRP (safety). Supplementary Material 13: Subgroup analysis for CRP (region). Supplementary Material 14: Subgroup analyses for overall effective rate (age). Supplementary Material 15: Subgroup analyses for overall effective rate (different control treatment). Supplementary Material 16: Subgroup analyses for overall effective rate (course of treatment). Supplementary Material 17: Subgroup analyses for overall effective rate (region). [file 2562590.f1.zip › 2562590.f1/Supplementary material9-Subgroup analysis for TC(Age).pdf]
